# Single cell characterization of myeloma and its precursor conditions reveals transcriptional signatures of early tumorigenesis

**DOI:** 10.1038/s41467-022-33944-z

**Published:** 2022-11-17

**Authors:** Rebecca Boiarsky, Nicholas J. Haradhvala, Jean-Baptiste Alberge, Romanos Sklavenitis-Pistofidis, Tarek H. Mouhieddine, Oksana Zavidij, Ming-Chieh Shih, Danielle Firer, Mendy Miller, Habib El-Khoury, Shankara K. Anand, François Aguet, David Sontag, Irene M. Ghobrial, Gad Getz

**Affiliations:** 1 Broad Institute of MIT and Harvard, Cambridge, MA USA; 2 CSAIL and IMES, Massachusetts Institute of Technology, Cambridge, MA USA; 3 Harvard Graduate Program in Biophysics, Cambridge, MA USA; 4 Department of Medical Oncology, Dana-Farber Cancer Institute, Boston, MA USA; 5 Harvard Medical School, Boston, MA USA; 6 Division of Hematology and Medical Oncology, Tisch Cancer Institute, Icahn School of Medicine at Mount Sinai, New York, NY USA; 7 Constellation Pharmaceuticals a MorphoSys Company, Cambridge, MA USA; 8 Cancer Center and Department of Pathology, Massachusetts General Hospital, Boston, MA USA

**Keywords:** Cancer genomics, Computational biology and bioinformatics, Tumour heterogeneity, Myeloma

## Abstract

Multiple myeloma is a plasma cell malignancy almost always preceded by precursor conditions, but low tumor burden of these early stages has hindered the study of their molecular programs through bulk sequencing technologies. Here, we generate and analyze single cell RNA-sequencing of plasma cells from 26 patients at varying disease stages and 9 healthy donors. In silico dissection and comparison of normal and transformed plasma cells from the same bone marrow biopsy enables discovery of patient-specific transcriptional changes. Using Non-Negative Matrix Factorization, we discover 15 gene expression signatures which represent transcriptional modules relevant to myeloma biology, and identify a signature that is uniformly lost in abnormal cells across disease stages. Finally, we demonstrate that tumors contain heterogeneous subpopulations expressing distinct transcriptional patterns. Our findings characterize transcriptomic alterations present at the earliest stages of myeloma, providing insight into the molecular underpinnings of disease initiation.

## Abstract (web summary)

Development of multiple myeloma is preceded by precursor conditions. Here, the authors use single cell RNA-sequencing of plasma cells from patients across disease stages to identify genomic signatures present even at the earliest stages of disease.

## Introduction

Multiple myeloma (MM) is a plasma cell (PC) malignancy residing in the bone marrow (BM)^1^. MM is almost always preceded by the precursor states monoclonal gammopathy of undetermined significance (MGUS) and smoldering multiple myeloma (SMM)^1–3^. However, the progression risk is highly heterogeneous, whereby certain patients progress quickly, while others never do. Patients with SMM exhibit progression rates of 10% per year, compared to just 1% for MGUS^4,5^. Currently, our ability to predict progression is mostly based on a few clinical parameters (e.g., M-spike, light chains, and percent tumor burden)^6,7^. Therefore, there is a need to further define molecular characteristics of patients who are at risk of progression. A thorough characterization of precursor cells and the state of the microenvironment in MGUS and SMM patients can help us distinguish the molecular mechanisms that underlie initial tumorigenesis versus later progression, predict which individuals are most at risk for progression, and identify potential targets for early therapeutic intervention.

Our understanding of genetic changes associated with disease progression and tumor evolution in MM is founded on studies that use clinical laboratory results and bulk analysis, including microarrays and DNA sequencing^8^. It has been shown that MGUS and SMM clones already harbor chromosomal alterations that define MM (translocations involving IgH or hyperdiploidy)^2,7^, and that progression to MM may be driven by the acquisition of secondary genetic events and mutational processes^9,10^. As such, stratification of SMM patients was recently updated by the International Myeloma Working Group to include cytogenetic abnormalities^11^. Additional studies showed that integration of events including *MYC* rearrangements, TP53 mono- and bi-allelic inactivation, and *RAS* mutants indeed help stratify patients by their risk of progression to active MM^12,13^.

At the RNA level, it is challenging to draw conclusions about the phenotype of precursor cells and the dynamics of malignant transformation from bulk RNA-sequencing studies^14^ due to low tumor purity (i.e., fraction of tumor cells in a sample) at the precursor stages. Recently, single cell studies of precursor conditions^15,16^ have allowed for characterization of these cells, but such datasets are still scarce and require careful computational analysis in order to glean insights from the limited number of abnormal cells present in biopsies from patients with precursor disease.

In this study, we generated and analyzed single cell RNA-sequencing (scRNA-seq) data from 29,387 PCs representing 26 samples from patients with MGUS, SMM, or MM as well as nine normal bone marrow donors (NBM). The single cell resolution of our data allowed us to isolate and compare abnormal and normal PCs from the same patient sample, even at precursor stages. We have previously analyzed the immune microenvironment of these same patients^17^ (Supplementary Table 1), and here we explore transcriptional changes within tumor cells as well as correlations between tumor and immune cell activity in our cohort. We identify abnormal cells from within a mixed sample, report our findings from a nuanced within-patient differential expression analysis approach, and employ automatic relevance determination non-negative matrix factorization (ARD-NMF)^18^ to highlight gene signatures that are active in our cohort and validated in external cohorts. Taken together, our study (i) presents a highly detailed and comprehensive view of the transcriptional transformation occurring in individual patients with myeloma and its precursor conditions, (ii) discovers gene expression signatures that are shared across patients with different driver events and at different stages of disease, and (iii) characterizes heterogeneity both between and within tumors.

## Results

### Single cell transcriptional profiles reflect driver events and reveal patient-specific patterns

**Fig. 1:**
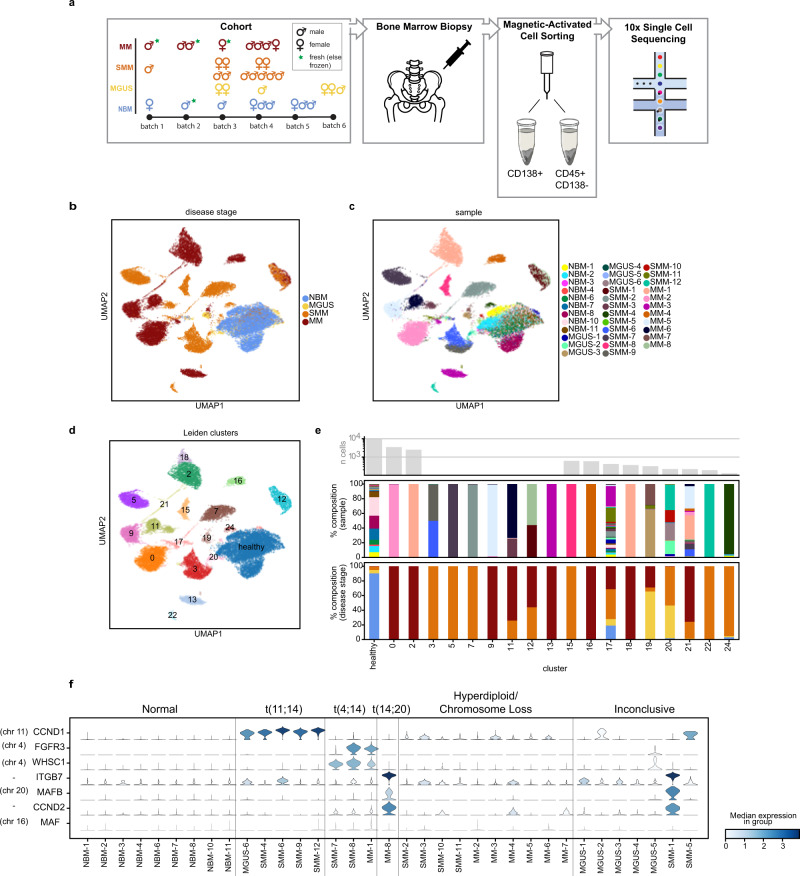
The landscape of normal and abnormal plasma cells at single cell resolution. **a** Overview of cohort and experimental setup, including the number of samples per disease stage, sex, sample preparation batch, and whether the sample was fresh or stored frozen prior to 10x sequencing. CD138+ bone marrow cell fractions were isolated and are analyzed in this study. **b**, **c** UMAP representation of plasma cells colored by disease stage (**b**) and sample ID (**c**). Cells similar in expression profile are placed nearby in this embedding. **d** Results of Leiden clustering of all cells. Seven clusters were merged to define a single cluster of healthy plasma cells. **e** Sample composition of Leiden clusters, by disease stage and sample ID (colors match the legends given in (**b**) and (**c**), respectively). The majority of clusters each consist of cells from a single sample. **f** Violin plots showing distribution of expression of genes commonly upregulated in patients with translocations (*y*-axis), along with annotations of the cytogenetic alterations detected in samples by clinical iFISH assay (top).

To investigate the gene expression dynamics of PCs at different stages of MM progression, we performed droplet-based scRNA-seq of 35 samples isolated from BM aspirates of patients with MGUS (*n* = 6), SMM (*n* = 12), newly diagnosed MM (*n* = 8), and nine healthy donors (NBM, *n* = 9; Fig. 1a; Supplementary Tables 2 and 3). One patient was biopsied both at the SMM stage and after progression to MM (SMM-1 and MM-8). Patients were followed for a median of 5.26 years (1921 days; range[1400, 5314]). Of the 18 patients with MGUS or SMM, 0/6 MGUS and 7/12 SMM patients were observed to progress to MM (Supplementary Table 2).

After filtering cells using standard quality controls, we analyzed a total of 29,387 single CD138+ PCs (~850 from MGUS, ~8.4 × 10^3^ from SMM, ~1.7 × 10^3^ from MM, and ~9 × 10^3^ from NBM). The number of CD138+ cells analyzed per sample ranged from 40 to 3414, with a median of 591 (Supplementary Table 3). Projecting cells onto a 2D Uniform Manifold Approximation and Projection (UMAP) plot, we observed that cells from our NBM samples grouped together, while the majority of cells from patients with precursor conditions and overt MM formed separate groups of cells (Fig. 1b, c).

Applying Leiden clustering^19^, we obtained 25 clusters of cells (Supplementary Table 4). Seven of these clusters represented healthy cells as determined by the majority of cells in these clusters coming from NBM samples and their overexpression of genes such as *CD27*. We merged these clusters into one “healthy” cluster (Fig. 1d). Of the remaining 18 clusters, 11 consisted almost exclusively of cells from a single sample each, reflecting the fact that normal variation between healthy individuals was minor compared to disease-associated expression changes (Fig. 1e). With a few exceptions (Supplementary Note 1), the clusters that represented multiple samples grouped cells with shared disease biology: cluster 12 contained two sequential samples from the same patient, cluster 21 contained proliferating abnormal cells from 15 patients across disease stages, and clusters 3 and 20 (together with cluster 24) represented all patients with a t(11;14) translocation (MGUS-6, SMM-4,6,9,12; Supplementary Fig. 1). We further confirmed that batch effects such as age, sex, and sample preparation were not driving clustering results (Supplementary Fig. 2).

Of note, our cohort included one patient, SMM-12, whose biopsy included two distinct subclones. Both subclones harbored a t(11;14) translocation and clonally expressed IgG kappa, suggesting that they descended from the same parental clone, but only one acquired a CD20+ phenotype (Supplementary Fig. 3a, b), a MM phenotype occurring in up to 22% of patients^20^. This falls in line with previous studies that have shown CD20+ myeloma cells to be correlated with translocation t(11;14)^21^. Cells from the CD20- subclone clustered together with cells from other t(11;14) samples in cluster 20, while cells from the CD20+ subclone clustered separately in cluster 22, suggesting large expression changes associated with the CD20+ phenotype. Indeed, comparing gene expression in the CD20+ vs. CD20− subclones, we found 455 differentially expressed genes (DEG) (|log(fold change)| *>* log(1.5); false discovery rate *q* < 0.1), with the top DEGs by *q*-value reflecting the B cell-like phenotype of these cells, including overexpression of *CD74*, *CD20* (also known as *MS4A1*), and HLA class II genes such as *HLA-DRA* and *HLA-DRB1* (Supplementary Fig. 3c; Supplementary Table 5).

To benchmark the performance of our experiment, we used interphase fluorescence in situ hybridization (iFISH) to identify large-scale structural genomic variants (Supplementary Table 2) and then inspected the expression levels of translocation target genes cyclin D1 (*CCND1*), MM SET domain (*MMSET/WHSC1*), fibroblast growth factor receptor 3 (*FGFR3*), MAF BZIP transcription factor (*MAF*), and MAF BZIP transcription factor B (*MAFB*), as well as cyclin D2 (*CCND2*) and Integrin Subunit Beta 7 (*ITGB7*), whose overexpression is also associated with translocations. MM cells from patients with iFISH-reported translocations exhibited overexpression of the respective target genes (Fig. 1f). In 4/7 samples whose iFISH results were inconclusive due to insufficient cell numbers, we were able to observe the overexpression of translocation partner genes or *CCND2* and *ITGB7*, indicating possible corresponding translocations. Our patient with sequential samples at SMM (SMM-1) and after progression to MM (MM-8) confirms the ability of RNA-sequencing to capture the cytogenetic phenotype even prior to iFISH; while this patient’s iFISH results were inconclusive in the sample taken during SMM, we were able to detect overexpression of *ITGB7*, *MAFB*, and *CCND2* at the transcriptional level, suggesting a t(14;20) translocation, which was later confirmed by iFISH after the patient’s progression to MM.

### In silico dissection of normal and abnormal cells within samples allows for characterization of disease even in samples with low tumor purity

**Fig. 2:**
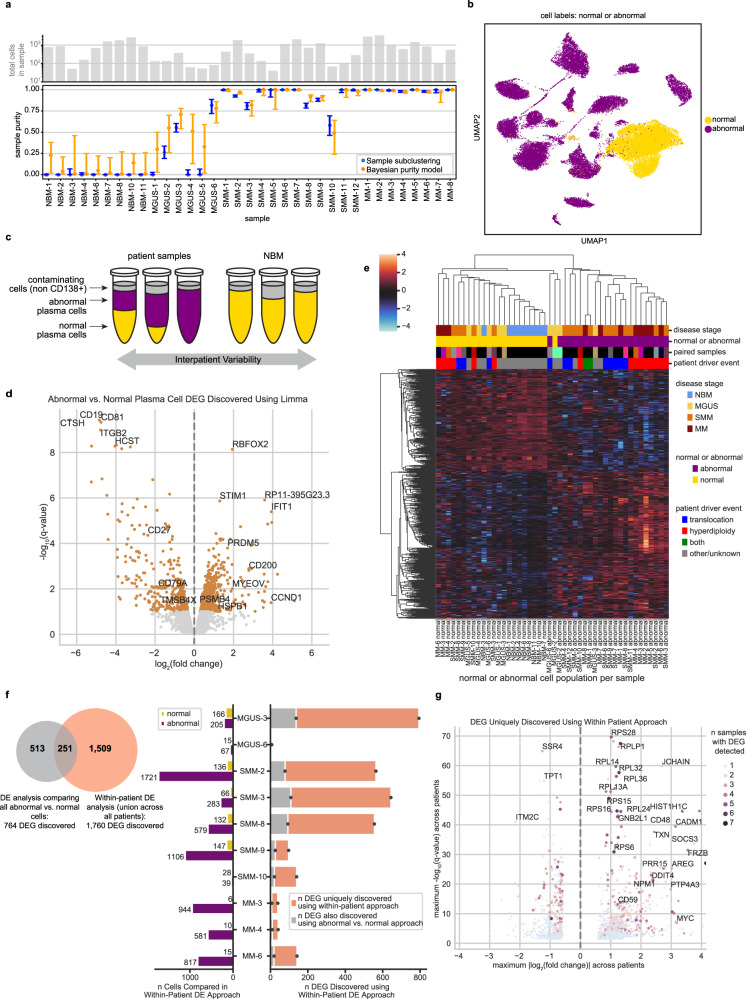
In silico dissection of transcriptional differences in normal and abnormal plasma cells within patient samples. **a** The number of cells (top) and estimated purity of each sample with 95% confidence intervals (bottom). Sample purity was estimated using two orthogonal methods: clustering of individual samples (blue; the fraction of cells labeled abnormal per sample is plotted) and our Bayesian hierarchical purity model (orange; the mode of posterior sample purity is plotted). Source data are provided as a Source Data File. **b** UMAP localization of individual cells labeled normal or abnormal. **c** Cartoon schematic of our differential expression analysis. We run two DE analyses: First, we compare all abnormal (purple) vs. all normal (yellow) cells using limma-voom. Next, we compare patients’ abnormal cells to their own normal cells, controlling for inter-patient variability. Samples with 100% normal or abnormal cells were excluded from the within-patient analysis. **d** Volcano plot of limma-voom DE results for abnormal vs. normal cell populations. Orange denotes genes with *q*-value < 0.1. The 4 most significantly up- and downregulated genes and other selected genes are annotated. **e** Pseudobulk expression of DEGs detected between abnormal and normal pseudosamples using limma-voom (z-scored per gene). Each column represents the normal or abnormal cells from a given sample. Color annotations denote disease stage (top), normal or abnormal (second), paired columns coming from the same sample (third; matching colors denote that columns correspond to the same sample; black denotes that there was no paired sample), and whether IgH translocation or hyperdiploidy was detected in that sample by iFISH (bottom). **f** Quantification of DEGs uniquely discovered using within-patient DE. The venn diagram represents the overlap of DEGs found using limma-voom and our within-patient DE approach. The bar plot describes the number of DEGs found per sample using within-patient DE (right side) and the number of abnormal and normal cells per sample (left side). **g** Volcano plot of 1760 DEGs uniquely discovered using our within-patient DE approach. The *y*-axis represents the maximum -log_10_(*q*-value) of the gene across samples included in the within-patient analysis, and x-axis represents the maximum log_2_(fold change). The color and size of a dot denote the number of samples for which that DEG was detected, with blue dots representing DEGs detected in just one sample.

One major benefit of studying precursor disease at single cell resolution is the ability to separate normal and abnormal CD138+ cells within each sample prior to downstream analyses. No individual marker genes can reliably distinguish these populations, but our full-transcriptome data enabled aggregation of an abnormal signal across many genes. To this end, we clustered the cells from each individual sample based on its highly variable genes (but excluding genes located in immunoglobulin loci), and then examined patterns of immunoglobulin and MM driver gene expression in each cluster to label the cluster as containing normal or abnormal cells (see Methods; Supplementary Fig. 4). To complement and validate this method, we also developed a Bayesian hierarchical model for estimating the tumor purity of each individual sample based only on the distribution of immunoglobulin light chain expression (see Methods; Supplementary Fig. 5). Comparing these results, we observed strong agreement between the two purity estimation methods (Fig. 2a). Our labels closely matched the Leiden clustering results (though not identically, highlighting the benefit of our curated labels), with 97% of cells we labeled as normal and <1% of cells we labeled as abnormal belonging to the healthy Leiden cluster (Fig. 2b).

Our purity results suggest that samples from patients with precursor conditions have a sizable fraction of normal PCs, as expected. On average, MGUS samples contained 73% normal cells and SMM samples contained 8%, compared to just 0.5% in MM. Furthermore, the variability of tumor purity values was also greater at early stages of disease. Whereas MM samples had consistently high tumor purity (range 0.98–1), we observed increasingly large variability in SMM (0.58–1) and MGUS (0–0.81), respectively. For our downstream analyses, we separated normal and abnormal cells within each sample and characterized them independently.

### Transcriptional differences between abnormal and normal cells across patients

We performed a differential expression (DE) analysis comparing abnormal and normal cells. To this end, we split samples into their abnormal and normal populations, which we refer to as “pseudosamples.” We compared abnormal pseudosamples to normal pseudosamples using limma-voom^22,23^ and found 764 DEGs (|log(fold change)| > log(1.5); false discovery rate *q* < 0.1; Supplementary Table 6).

In addition to genes known to be important for MM biology like *CCND1*^8^ (upregulated), *CD27*^24–26^ (downregulated) and *TMSB4X*^27^ (downregulated), we also found other strongly regulated genes whose connection to myeloma is less well characterized. The top 4 upregulated DEGs by *q*-value included *RBFOX2*, *STIM1*, a transmembrane protein that mediates store-operated calcium entry and is of interest in multiple cancers^28–30^, *IFIT1*, and the long non-coding RNA *RP11-395G23.3*. The top 4 downregulated genes included *CD19*, a B cell lineage marker gene which has been explored as a therapeutic target in MM^31,32^, *CTSH*, *CD81*, which regulates *CD19*^33^ and has been shown by flow cytometry to be downregulated in MM and precursor conditions^34^, and *ITGB2*. We also observed upregulation of *PSMB4* and *HSPB1*, which are associated with the proteasome (Fig. 2d).

Unsupervised clustering of the pseudosamples based on their expression of these 764 DEGs showed good separation of abnormal and normal samples, as expected, and revealed that hyperdiploid patients exhibit especially high expression of these upregulated DEGs and tend to cluster together. Abnormal samples did not cluster by disease stage, underscoring the fact that many of these DEGs are altered in both myeloma and precursor samples. All abnormal populations from SMM samples clustered together with the myeloma cells, while from MGUS, one abnormal pseudosample clustered with myeloma cells and two clustered with the normal cells. The two abnormal samples that clustered together with normal cells came from MGUS patients with very low numbers of abnormal cells detected (*n* = 35 and 67 cells for MGUS-2 and MGUS-6, respectively). Thus, we could not conclude that the MGUS phenotype is similar to that of normal cells, since the averaged gene expression in those pseudosamples is inherently noisy. Abnormal cells from MGUS-3 (*n* = 205), on the other hand, clustered together with other abnormal samples (Fig. 2e).

Interrogating MSigDB hallmark genesets, we found that pathways related to E2F targets, Notch signaling, G2M checkpoints, interferon alpha response, and Wnt/beta-catenin signaling are differentially enriched in abnormal samples compared to normal (*t*-test *q* < 0.1; Supplementary Fig. 6a). To investigate which pathway changes are initiated at early stages of disease, we compared pathway enrichment results specifically between MGUS and normal samples and found that the Wnt/beta-catenin pathway is already upregulated (*t*-test *q* = 0.08). Individual upregulated genes from the Wnt/beta-catenin pathway include *DKK1*, *KAT2A*, and *TP53* (limma-voom abnormal vs. normal *q* < 0.025). Low sample size (*n* = 3) for abnormal MGUS samples may have hindered our power to discover other pathways that are already differentially enriched in MGUS vs. normal.

This DE analysis provides a general view of genes whose expression is consistently altered in disease, but it does not allow us to discover genes whose expression may be altered in just a small subset of patients in our cohort. Additionally, while normal cells are more similar to each other than abnormal cells are (Fig. 1b, c), inter-patient differences still exist among them (Supplementary Fig. 7). Thus, this analysis suffers from both high variance due to the small number of normal samples and confounding effects due to non-disease-related differences between individuals that contributed healthy bone marrow and tumor samples. We address these limitations with the following analysis.

### Within-patient abnormal vs. normal cell comparisons highlight inter-patient heterogeneity and patient-specific disease characteristics

To account for the limitations of the DE analysis described above, we leveraged samples containing mixtures of normal and abnormal plasma cells to perform a “within-patient” characterization of the disease. For each patient, we compared their abnormal plasma cells to their own healthy plasma cells (Fig. 2c). This allowed us to specifically characterize the unique transcriptional profiles of individual tumors, which may not be shared across patients, without introducing the confounding effects that would arise from comparing tumor cells to normal cells from other healthy donors.

Of our eleven patients with both abnormal and normal cell populations, ten had significant DEGs detected between these populations (|log(fold change)| > log(1.5); false discovery rate *q* < 0.1). Overall, this method identified 1760 DEGs (Supplementary Table 7), 1509 of which were not found in the general abnormal vs. normal DE analysis described above (Fig. 2f). We found DEGs that are unique to individual patients (1323 genes) as well as genes recurrently affected across patients, such as *CD27* (upregulated in eight patients), *CD79A* (upregulated in seven patients), and *RPL25* (downregulated in seven patients). Many DEGs were shared across samples from multiple disease stages (Supplementary Fig. 6b).

We next highlight some of the genes that were discovered in our within-patient DE analysis, but not in the general DE analysis described earlier (Fig. 2g). For example, within-patient DE identified significant upregulation of *FGFR3* and *WHSC1* in our patient with t(4;14). The general abnormal vs. normal cell comparison was not powered to identify this upregulation, since the translocation only occurred in a single patient in our cohort. Additionally, we discovered upregulation of *GNB2L1* (also known as *RACK1*; up in SMM-2, SMM-3, MM-6), a known oncogene in other cancers^35,36^ that has recently been reported to be upregulated in myeloma cell lines^35,36^, but not yet in clinical samples. Among upregulated genes, we also found the histone gene *HIST1H1C* (MGUS-3, SMM-2, SMM-3, SMM-8, MM-6), the cell surface markers *CD48* (MGUS-3, SMM-8) and *CD59* (MGUS-3, SMM-2, SMM-3, SMM-8, SMM-10), and the proto-oncogene *MYC* (MGUS-3, SMM-2, MM-6) (Fig. 2g; Supplementary Fig. 6c). We observed downregulation of *SSR4* (SMM-2, SMM-3, MM-3), associated with translocation of proteins across the endoplasmic reticulum, and *TPT1* (MGUS-3, SMM-8), a regulator of cell growth and proliferation. *ITM2C*, which has been reported for its expression on MM cells^37,38^, was upregulated in some samples (MGUS-3, SMM-2, SMM-3, SMM-8) but downregulated in others (MGUS-6, SMM-9). While higher expression of *ITM2C* has been reported in patients with t(4;14) vs. without^39^, we cannot conclude this from our data, as *ITM2C* was variably expressed in our three samples with t(4;14) (SMM-7, SMM-8, MM-1; Supplementary Fig. 6d). Ribosomal proteins such as *RPS28* (SMM-2, SMM-3, SMM-10, MM-3, MM-6), *RPLP1* (MGUS-3, SMM-2, SMM-3, SMM-10, MM-3, MM-4), *RPL14* (MGUS-3, SMM-2, SMM-3, SMM-10, MM-6), and others were recurrently upregulated, specifically in patients with hyperdiploidy. Although these ribosomal protein genes were upregulated in multiple samples, other samples had expression levels similar to those of NBM samples (Supplementary Fig. 6d), possibly explaining why they were only detected using within-patient DE.

### NMF discovers gene signatures that capture transcriptional programs

While our within-patient DE analysis allowed us to discover gene signatures in individual tumors, we next employed a method to discover gene signatures active in individual cells across our cohort, even if only in a small subset of cells in a tumor, and to characterize signature activity at the single cell level across disease stages.

**Fig. 3:**
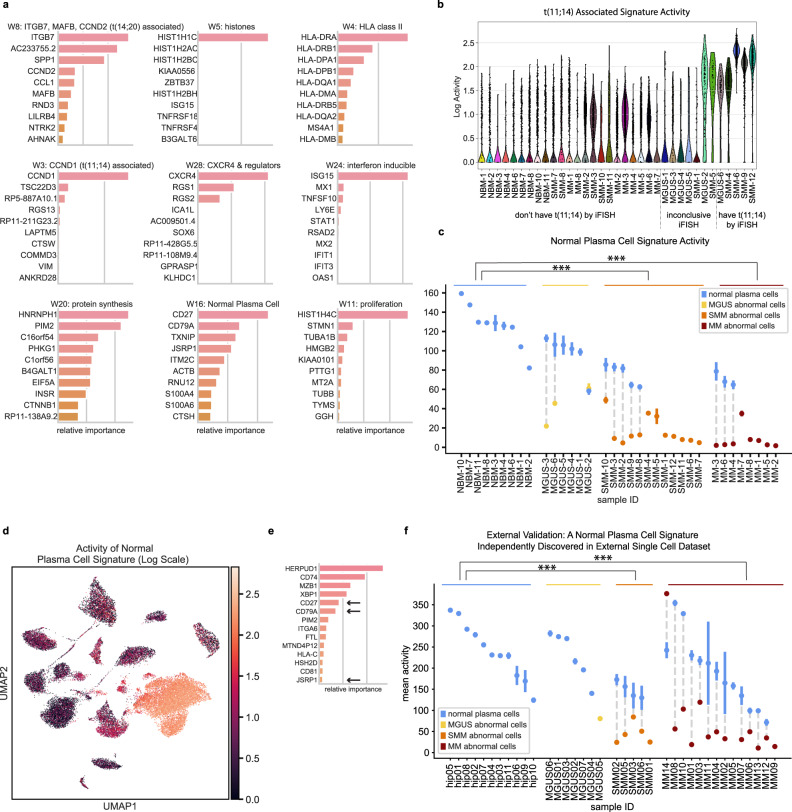
Bayesian non-negative matrix factorization uncovers gene signatures which capture myeloma cell biology across disease stages. **a** Top genes for nine representative gene signatures. The importance score, plotted on the x-axis, is based on both the strength of the gene’s contribution to the signature and its specificity to the signature (see Methods). **b** A signature with top contribution from *CCND1* is discovered and is most active in samples with t(11;14), as expected. **c**, **d** We discover a ‘normal plasma cell signature’ that is active in normal plasma cells across disease stages and downregulated in abnormal cells from MM and precursor conditions. We visualize this signature’s activity by showing its mean activity ± s.e.m. for the normal and abnormal populations within each sample (**c**) and on a UMAP plot (log scale) (**d**). Mean activities were compared between groups, with *** denoting *q* < 0.001 for group differences (abnormal cells from SMM (*n* = 12) and MM (*n* = 8), respectively, significantly differed from NBM (*n* = 9)). **e** Validation on external dataset: our NMF algorithm run on external CD138+ single cell data from MGUS, SMM, MM and healthy donors independently discovers a gene signature similar to our normal plasma cell signature, with shared top genes *CD27*, *CD79A*, and *JSRP1*. **f** After labeling cells in that dataset as normal or abnormal, we discover that this signature follows the same pattern as in our data, with high activity in normal cells and a significant decrease in activity in abnormal cells across disease stages. Mean activities ± s.e.m. across cells in normal and abnormal portions of samples are shown, with *** denoting *q* < 0.001 for group differences (abnormal cells from SMM (*n* = 5) and MM (*n* = 13), respectively, significantly differed from NBM (*n* = 11)). Source data are provided as a Source Data File.

Using our ARD-NMF method^18,40^, we decomposed the gene expression profiles across all plasma cells in our cohort into 28 gene signatures (Supplementary Table 8). Because we were most interested in highlighting signatures associated with disease biology rather than patient-specific effects, we removed signatures that were only active in a single patient. Similarly, since our goal was to find groups of genes with shared activity patterns, we did not focus our downstream analyses on signatures that only represented the expression of a single gene. After removing these “patient-specific” and “single-gene” signatures, we retained 15 gene signatures and examined the top genes from each signature to identify its underlying biological mechanism (Fig. 3a; Table 1). We confirmed that signature activities did not correlate with batch variables (see Methods).

**Table 1:** 15 gene expression signatures discovered using ARD-NMF (“patient-specific” and “single-gene” signatures are not included; see Supplementary Table 8 for the full list of signatures)

Signature	Biological description	Top genes
W3	t(11;14) associated	CCND1, TSC22D3, RP5-887A10.1, RGS13
W4	HLA class II	HLA-DRA, HLA-DRB1, HLA-DPA1, HLA-DPB1
W5	Histones	HIST1H1C, HIST1H2AC, HIST1H2BC, KIAA0556
W8	t(14;20) associated	ITGB7, AC233755.2, SPP1, CCND2
W9	Extracellular signaling	LGALS1, VIM, ACTB, S100A6
W11	Proliferation	HIST1H4C, STMN1, TUBA1B, HMGB2
W16	Normal plasma cell	CD27, CD79A, TXNIP, JSRP1
W20	Protein synthesis	HNRNPH1, PIM2, C16orf54, PHKG1
W24	Interferon inducible	ISG15, MX1, TNFSF10, LY6E
W28	CXCR4 & regulators	CXCR4, RGS1, RGS2, ICA1L
W1	Unknown	JUNB, ZFP36, NFKBIA, IER2
W6	Unknown	DUSP4, GADD45A, BTG2, LAMP5
W14	Unknown	KLF6, TSC22D3, ANKRD28, KLF2
W26	Unknown	HLA-A, ITM2C, PRR15, ACTB
W27	Unknown	NEAT1, DDX17, ANKRD12, FOXO3

A number of the NMF signatures represented subtypes of myeloma that have previously been reported; for example, we found a signature of proliferation similar to that reported by Zhan et al.^41^ and Broyl et al.^42^, a *CCND1*-related signature which is differentially active in samples with t(11;14) (Fig. 3b), and a signature composed of *MAFB, CCND2*, and *ITGB7*, which is active in samples with t(14;20).

We additionally discovered signatures that captured less well-characterized disease biology common to multiple samples in our cohort. For example, we found a signature with top genes *CXCR4*, which plays a role in normal plasma cell development and has also been implicated in MM progression^43,44^, and *RGS1* and *RGS2*, which are regulators of G Protein signaling that may regulate the CXCR4-CXCL12 axis^45^. We additionally found signatures that represent the activity of histone genes, interferon (IFN)-inducible genes, and genes involved in protein synthesis, among others.

### Gene signature activity correlates with disease stage and microenvironment

For each gene signature, we tested whether its activity level varied between abnormal and normal cell populations or with disease stage, and discovered that three signatures had significantly different activity levels (Kruskal–Wallis *q* < 0.1 and Dunn’s *q* < 0.1). As expected, we found that the t(11;14)-related signature is differentially active in abnormal cells from samples with the corresponding translocation compared to NBM cells. We found two additional signatures whose activity was significantly correlated with disease, described in detail below.

#### Abnormal cells across disease stages share universal downregulation of gene signature seen in normal PCs

We discovered a “normal plasma cell signature” that is downregulated in abnormal cells at all stages of disease (*q* = 3.3 × 10^−5^ and 1.0 × 10^−6^ for SMM and MM vs. NBM, respectively; while the same trend is observed in MGUS abnormal cells vs. NBM, it did not reach significance, likely due to low number of MGUS cases with sufficient abnormal cells). This signature robustly characterizes the normal bone marrow plasma cells in our data set (Fig. 3c, d), and highlights genes that are downregulated in the abnormal cell portions of samples at all disease stages. The top genes in this signature include *CD27* and *CD79A*, which are associated with the B cell lineage, as well as *JSRP1*, *CTSH*, *HCST*, and *RNU12* (Fig. 3a; Supplementary Fig. 8a). Other canonical B cell markers (*CD20*, *BCL6, PAX5*, and *E2F1)* were not expressed on the normal PCs, suggesting this observed downregulation is not due to B cell contamination among the normal cells in our cohort (Supplementary Fig. 9a, b). Given the low tumor purity during early precursor conditions, this phenotype would be obscured at early disease stages in bulk samples; analysis at a single cell resolution, however, reveals that this healthy plasma phenotype is significantly downregulated in abnormal cells as early as the MGUS stage (Fig. 3c). Indeed, for our patient with serial samples at the SMM and MM stages (SMM-1 and MM-8), we find similarly low levels of signature activity at these two timepoints, underscoring the fact that this phenotype is lost at early stages of disease and remains low as the disease progresses (Fig. 3c). Interestingly, the activity of this signature in normal cells also trends downward with increasing disease stage (Jonckheere-Terpstra test *p* = 1.3 × 10^−5^).

The individual top genes on this signature are also downregulated in patients’ abnormal cells compared to normal cells (Supplementary Fig. 8b). The notable exception is our CD20+ sample, SMM-12: while *CD27* is upregulated in abnormal cells vs. normal cells in this sample, the NMF normal plasma cell signature nonetheless has low overall activity (Fig. 3c), demonstrating the universal loss of this signature across tumors with different phenotypes.

#### Validation of normal plasma cell signature in independent datasets

To validate our findings, we ran the ARD-NMF algorithm on single cell data from Ledergor et al.^15^ and recovered a similar signature with top genes *CD27*, *CD79A*, and *JSRP1*. This signature, too, is strongly downregulated in abnormal cells at all disease stages (*q* = 2.4 × 10^−4^ and 1.5 × 10^−5^ for SMM and MM vs. healthy donors, respectively; only one MGUS sample had abnormal cells, and it too appears to be downregulated; Fig. 3e, f). As additional validation in bulk data, we estimated the activity of our normal plasma cell signature in bulk RNA sequencing from newly diagnosed MM patients in the Multiple Myeloma Research Foundation’s (MMRF) CoMMpass dataset, and also estimated tumor purity in these samples (see Methods). We found a significant negative correlation between signature activity and tumor purity, further supporting this signature as a marker of normal plasma cells (Supplementary Fig. 8c).

#### Interferon-inducible signature upregulated in tumor and microenvironment

**Fig. 4:**
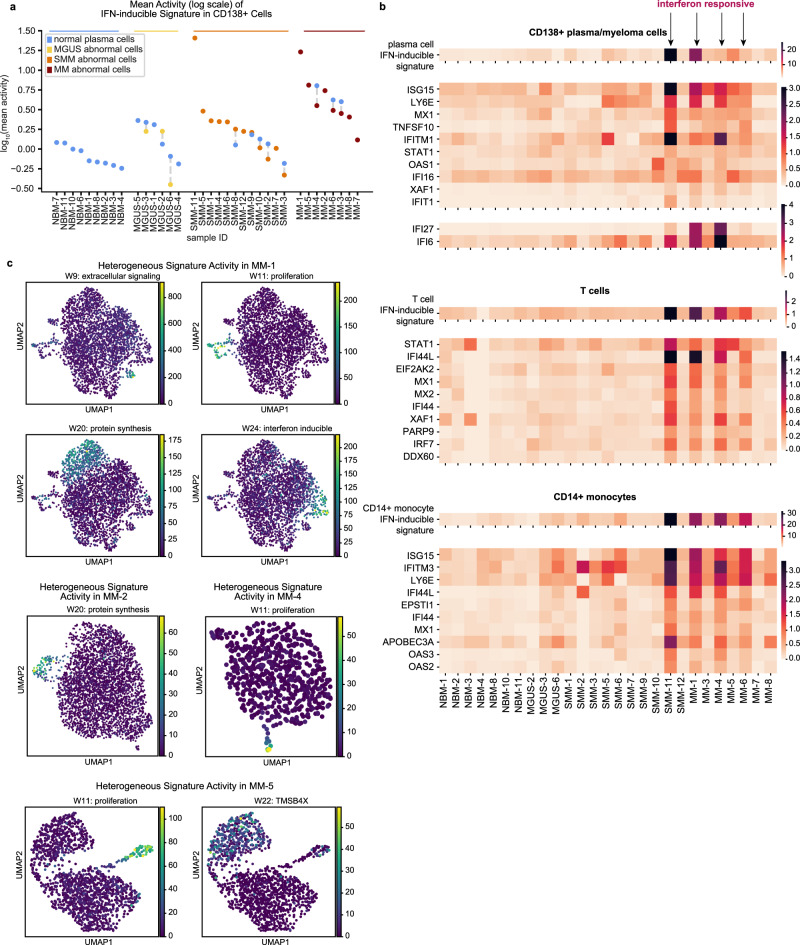
IFN-inducible signature is correlated between CD138+ and microenvironment cells, and gene signatures exhibit intratumor heterogeneity. **a** Mean activity ± s.e.m. of CD138+ IFN-inducible signature across normal and abnormal plasma cell populations. Both normal and abnormal plasma cells exhibit significantly increased activity of the interferon-inducible signature in MM vs. NBM (*q* = 5.2 × 10^−3^ and *q* = 3.2 × 10^−4^, resp.). Source data are provided as a Source Data File. **b** Mean activity per sample of IFN-inducible signature discovered in CD138+ cells (top), T cells (middle) and CD14+ monocytes (bottom). Mean expression levels for the ten genes with the highest values in the W matrix for each signature are also shown. Expression of additional interferon-inducible genes IFI27 and IFI6 is shown for CD138+ samples (see Supplementary Note 2). CD138+ samples from patients were limited to abnormal cells before calculating means. NMF signature results and expression data for T cells and monocytes were taken from Zavidij et al.^17^. **c** Subpopulations within patient tumors heterogeneously express gene signatures. Cells from a given MM sample were projected onto a UMAP plot based on expression of highly variable genes, and colored by the activity level of NMF signatures determined to be heterogeneously expressed in that sample (see Methods).

We discovered a signature enriched for IFN-inducible genes, such as ISG15 ubiquitin like modifier (*ISG15*), MX Dynamin Like GTPase 1 (*MX1*), and Interferon Induced Protein With Tetratricopeptide Repeats 1 and 3 (*IFIT1* and *IFIT3*)^46^ (Fig. 3a). Notably, this signature is significantly upregulated in both normal and malignant populations from overt MM patients compared to NBM (*q* = 5.2 × 10^−3^ and *q* = 3.2 × 10^−4^, respectively; Fig. 4a). This upregulation is specific to malignant disease, i.e. the signature is not significantly upregulated in precursor conditions (one patient, SMM-11, is an outlier with very high activity). Further, a previous study^17^ discovered similar IFN-inducible signatures when running ARD-NMF on T cells and CD14+ monocytes from the microenvironments of these same patients’ tumors. We found that the patients who had high IFN-inducible gene signature activity in their T cells and monocytes also had high activity of this signature in their CD138+ cells (Fig. 4b). T cell and monocyte markers were not expressed in CD138+ cells, suggesting this correlation is not due to cell type contamination (Supplementary Fig. 9c).

### Tumors contain transcriptionally heterogeneous cell subpopulations

The NMF approach to signature discovery allows us to find groups of genes with shared activity in single cells, and thus not only to examine how signature activity varies between samples and disease stages, but also between subpopulations of abnormal cells within a single sample. Indeed, we find that tumors are heterogeneous, with subpopulations of cells expressing distinct subsets of the NMF gene signatures we discovered (see Methods). For example, considering our samples from myeloma patients, in MM-1, disjoint subsets of cells expressed the IFN-inducible, proliferation, extracellular signaling and protein synthesis signatures; this is discernible by coloring MM-1’s cells either by the activity level of these signatures (Fig. 4c; Supplementary Fig. 11) or the top genes from these signatures (Supplementary Fig. 10a). Similarly, MM-2 contains a subset of cells with high expression of the protein synthesis signature, MM-4 contains a subset of proliferating cells, and MM-5 contains a subset of proliferating cells as well as cells with varying activity of signature 22 (a single-gene signature representing the expression of *TMSB4X*) (Fig. 4c; Supplementary Figs. 10b–d, 11). The NMF signatures that were found to exhibit intratumor heterogeneity in multiple samples were those relating to HLA class II genes (heterogeneous in two samples), extracellular signaling genes (heterogeneous in two samples), proliferation genes (heterogeneous in eight samples), protein synthesis genes (heterogeneous in two samples) and IFN-inducible genes (heterogeneous in two samples).

## Discussion

Early identification of patients with precursor conditions at risk of progressing to overt MM could allow for early therapeutic intervention. However, the current risk criteria used to identify high risk precursor patients who would most benefit from treatment are mostly based on clinical parameters such as M spike, light chains or percent tumor burden^6^. Therefore, elucidation of the molecular transformation that occurs at early tumorigenesis and later at high risk SMM before disease progression is critical for developing informed criteria for patients who would benefit from early intervention and targets that may be exploited for therapeutics^9,10,47^.

Here, we leveraged single-cell RNA sequencing to overcome the challenges of characterizing the transcriptomics of these low burden precursor states. While some patients at early stages of disease had low disease burden, such that their driving cytogenetic translocations could not yet be detected by iFISH in the clinic, we demonstrate that scRNA-seq is sensitive enough to already reveal their underlying cytogenetic changes. This result highlights the possibility of using RNA sequencing to detect changes in patients’ bone marrow plasma cells earlier than methods currently used in the clinic^48^.

Precise labeling of normal and abnormal cells in each sample revealed low tumor purity in samples from earlier disease stages, even when subsetting to only CD138+ cells. This suggests that conclusions drawn from bulk studies of precursor conditions are likely influenced by heavy contamination from normal plasma cells. For example, Chng et al.^14^ concluded that their “MYC activation signature” is upregulated in a subset of myelomas, but not in MGUS. While it is possible that MYC activation really did not occur in their MGUS samples, we find *MYC* and some of their MYC activation signature genes to be significantly upregulated in two precursor patients in our cohort (MGUS-3 and SMM-2), as well as in two MM patients (MM-5 and MM-6). The upregulation of *MYC* in MGUS is clinically relevant, as it is associated with tumor aggression, poor clinical outcomes, and potentially with disease progression^12,13,49^. Both the low tumor purity in MGUS and the potential rareness of this phenotype among MGUS patients would have made this difficult to discover without single cell data, the ability to distinguish normal vs. abnormal cells, and our within-patient DE analysis.

Through our isolation of abnormal cells, we found that patients with early precursor conditions already exhibit transcriptomic alterations seen in overt MM. As one important example, we identified a signature present in normal plasma cells but uniformly lost at all stages of MM progression. *CD27*, one of the top genes of this signature, has been previously discussed in MM literature, but has been reported to have variable expression in myeloma cells, increased expression in MGUS, and a correlation with prognosis^24–26^. Our data shows significant downregulation of *CD27* compared to normal plasma cells as early as the MGUS stage (Supplementary Table 7; Supplementary Fig. 6c). Although we observe a trend of decreased *CD27* expression in MM compared to SMM (Supplementary Fig. 6c), it raises the question of the extent to which previous results were confounded by increasing tumor purity as the disease progresses. This would need to be tested in a larger cohort with single cell data.

In addition to *CD27*, abnormal plasma cells had lower expression of another mature B cell marker, *CD79A*, as well as decreased enrichment of immune pathways, such as complement pathway (including decreased expression of complement receptor 2, *CR2*, also known as *CD21*). Similarly, other studies found absence or low levels of B cell surface markers CD19, CD27, and CD45 on abnormal cells compared to normal plasma cells^24,50^. Our study extends the characterization of matched abnormal and normal plasma cells to the whole transcriptome. It also supports the hypothesis that the loss of B cell immune functionality, as assessed by gene expression programs and cell surface protein expression, is an early step in the generation of tumor plasma cells.

When probing pathway level transcriptional changes in abnormal cells, we found aberrant expression of Wnt pathway members including overexpression of *DKK1* in abnormal cells of precursor myeloma (Supplementary Fig. 4c). DKK1 is secreted by myeloma cells and is associated with the presence of osteolytic lesions through inhibition of osteoblast differentiation^51,52^. Given that many cases of MGUS also have osteoporosis and osteopenia, this may indicate that Wnt dysregulation and *DKK1* overexpression are associated with early osteopenia in those patients and are potentially predictors of the development of osteolytic lesions.

When investigating connections between the phenotype of PCs and dynamics in the tumor immune microenvironment, we discovered that patients who exhibit upregulation of an IFN-inducible signature in their tumor cells exhibit this same phenotype in their normal bone marrow PCs, as well as in T cells and monocytes in their tumor microenvironment. This suggests that interferon signaling in myeloma cells, which has been reported previously^53^, may be a response to a common stimulus in the microenvironment that affects multiple cell types, including normal plasma cells. Further work is needed to investigate the potential common mechanisms driving the upregulation of interferon signaling across these cell types.

The single cell resolution of our data provides insight into the heterogeneity of signatures within patient samples. For example, while the proliferation signature has previously been reported in bulk studies as characterizing a distinct subset of patients^41^, our data reveal that in fact, only a subset of cells from any given patient exhibit a proliferative signature. In addition, these proliferating cells may be found in patients that harbor the driver mutations previously used to characterize patients that belong to the non-proliferative subtypes.

Finally, our within-patient DE analysis points to potential therapeutic targets that are present in subsets of patients not only in MM, but also in earlier disease stages, and which can be selected for functional validation. We found that select patients exhibit upregulation of genes associated with the proteasome, which may correlate with sensitivity to bortezomib and antitumor immune response^54^. While certain proteasome genes were identified in our general abnormal vs. normal DE analysis (*PSMB4*, *HSPB1*), we discovered upregulation of additional proteasome genes in select patients using our within-patient DE approach (e.g. *PSMA4*, *PSMD14*, *PSMD11*, *PSMC6*, and *PSMA1* in MGUS-3 and SMM-8), painting a much fuller picture of proteasome-related gene dysregulation in those samples. Additionally, this approach allowed us to detect that *CD59*, a complement inhibitor whose expression has been associated with resistance to daratumumab in myeloma^55^ and to anti-CD20 therapies in B cell malignancies^56^, was significantly upregulated in five patients, including those with precursor conditions. In a similar vein, we discovered upregulation of *CD48*, which has been nominated as a drug target in MM^57^, in abnormal cells from MGUS and SMM. Our identification of patient-specific transcriptional changes as early as MGUS paves the way for future work exploring personalized treatment approaches prior to malignant disease.

In summary, our work used single-cell RNA sequencing to overcome the low fraction of abnormal plasma cells in early precursor disease and uncover early-occurring transcriptional changes which could not be accurately described in prior bulk sequencing studies. We identified commonalities between MGUS and overt MM, such as the loss of our reported normal plasma cell signature. We elucidated patient-specific transcriptional changes, laying a foundation for the development of precision medicine approaches for treating MM and potentially intercepting precursor conditions prior to progression.

## Methods

### Patient samples and cell preparation

BM samples from patients with MGUS, SMM or MM, were collected as approved by the Dana-Farber Cancer Institute Institutional Review Board (protocols 14–174, 07–150, or 11–104). Informed consent was obtained from all patients in accordance with the Declaration of Helsinki protocol (fifth revision from 2000 with Clarifications of Articles 29, 30 (20022004), and the most recent iteration from 2013). MGUS and SMM patient samples were collected for a clinical trial, clinicaltrial.gov identifier NCT02269592. Samples from healthy donors were purchased from the company AllCells. CD138+ BM cell fractions were isolated using magnetic-activated cell sorting technology (Miltenyi Biotec). Selected cells were either viably cryopreserved in dimethylsulfoxide at a final concentration of 10% or used immediately for scRNA-seq.

### Sequencing library construction using the 10x Genomics platform

Frozen BM cells were rapidly thawed, washed, counted and resuspended in PBS and 0.04% bovine serum albumin to a final concentration of 1000 cells per µl. The Chromium Controller (10x Genomics) was used for parallel sample partitioning and molecular barcoding. To generate a single-cell Gel Bead in Emulsion, cellular suspensions were loaded on a Single Cell 3′ chip together with the Single Cell 3′ Gel Beads, according to the manufacturer’s instructions (10x Genomics). scRNA-seq libraries were prepared using the Chromium Single Cell 3′ Library Kit v.2 (10x Genomics). Fourteen cycles were used for the total complementary DNA amplification reaction and for the total sample index PCR. Generated libraries were combined according to Illumina specifications and paired-end sequenced on HiSeq 2500/4000 platforms with standard Illumina sequencing primers for both sequencing and index reads; 100 cycles were used to sequence Read1 and Read2.

### Preprocessing of scRNA-seq data

Sample demultiplexing, barcode processing, alignment to the human genome (hg38) and single-cell 3′ gene counting was performed using the Cell Ranger Single-Cell Software Suite v.2.0.1. Cells called by Cell Ranger were further filtered to those with <15% mitochondrial expression, >200 genes covered, <50,000 total unique molecular identifiers (UMIs), and <4000 total genes detected. Log-normalized expression values were calculated as:

1 $${{{{e}}}}_{{{{g}}},{{{c}}}}={{\log }}\left(\frac{{10}^{4}}{{{{{N}}}}_{{{{c}}}}}{{{{n}}}}_{{{{g}}},{{{c}}}}+1\right)$$

for a cell *c* with *N*_*c*_ total UMIs from genes (excluding genes that accounted for >20% of UMIs in any cell), with *n*_*g,c*_ UMIs mapping to gene *g*. Except where noted, “expression” refers to log-normalized expression.

### Gene selection

For downstream analyses (PCA, UMAP, Leiden clustering, differential expression, NMF), we removed genes located in IGH, IGL, or IGK loci (based on the GRCh38 reference), since these are expected to be upregulated and clonally expressed in abnormal cells, dominating other transcriptional disease signatures of interest. Sex genes *XIST* and *RPS4Y1* (the two genes with the greateset absolute fold changes when comparing gene expression in male vs. female samples in our cohort) were also removed prior to PCA, UMAP, clustering, and NMF, so as not to separate samples based on the sex of the patient but rather based on disease biology. Highly variable genes were selected based on log-normalized expression data using the highly_variable_genes function in Scanpy^58^ (version 1.7.1) with default parameters and max_mean = 4, except where indicated otherwise.

### Removing non-CD138+ cell populations

To remove cells incorrectly sorted during bead selection, we first performed coarse clustering of all cells sorted as CD138+. We centered and scaled the data, clipping the resulting values to a maximum of 10, calculated highly variable genes, projected the expression of highly variable genes onto its first 14 principal components, and computed Leiden clusters (resolution = 1.5), all using the Scanpy^58^ (version 1.7.1) package with default parameters except as specified. We chose this high resolution for clustering as our goal was to find and remove even small clusters of contaminating non-CD138+ cells. Using expression of known cell type markers, we identified and removed clusters of cells containing non-CD138+ immune cells, red blood cells, and cells from the extracellular matrix.

### Leiden clustering of CD138+ cells

After removing contaminating cell types, we reprocessed our data prior to downstream analyses. Despite removing clusters of red blood cells, we still detected ambient contamination of hemoglobin genes in some samples, and thus we regressed out a "hemoglobin score," computed as the mean of log-normalized expression of hemoglobin genes. We then recomputed highly variable genes, re-centered and scaled the data, clipping the resulting values to a maximum of 10, projected the expression of highly variable genes onto its first 14 principal components, and computed Leiden clusters (resolution = 1.5), all using the Scanpy package^58^ (version 1.7.1) with default parameters except as specified. Here, we again chose a high resolution for clustering in order to detect even small clusters of unique cell types. We merged seven clusters which were all determined to contain healthy cells based on the majority of cells in these clusters coming from NBM samples, their overexpression of genes such as *CD27*, and their co-localization on a 2D UMAP embedding plot.

### Bayesian model for sample purity estimation

We developed the following hierarchical Bayesian model to automatically estimate ρ, the purity of a sample, based on the number of cells expressing the kappa immunoglobulin gene and the total number of cells in the sample. This model is based on the rationale that since abnormal cells in a sample are descended from a single B-cell progenitor with a specific V(D)J rearrangement, they will uniformly express either kappa or lambda immunoglobulins. In contrast, the normal plasma cells are a highly diverse group of cells with a ratio of kappa versus lambda immunoglobulins reflective of the relative frequencies of these rearrangements. By analyzing our NBM samples, we observe that the fraction of normal cells with kappa rearrangements are similar across individuals, albeit with some variance which we model with a truncated normal distribution (between 0 and 1). The measured frequency of cells with kappa vs. lambda rearrangements is a function of the mixture of cells originating from these two distributions. We can therefore use the observed counts in each sample to calculate the probability that a sample is composed of a given proportion of normal and abnormal cells (i.e. estimate sample purity).

Specifically, we assume the following generative model,

$${\kappa }_{{{{n}}}}	 \sim {{{{{\rm{Truncated}}}}}}\,{{{{{\rm{Normal}}}}}}(\mu,{\sigma }^{2},0,1) \\ {\kappa }_{{{{t}}}}	 \sim {{{{{\rm{Bernoulli}}}}}}(0.5) \\ \rho	 \sim {{{{{\rm{Beta}}}}}}(1,1)\\ {{{p}}}	=\rho * {\kappa }_{{{{t}}}}+(1-\rho )* {\kappa }_{{{{n}}}}\\ {{{{n}}}}_{\kappa }	 \sim {{{{{\rm{Binomial}}}}}}({{{N}}},{{{p}}})$$

where *κ*_*n*_ is the proportion of kappa cells among normal cells in a sample, *κ*_*t*_ is the proportion of kappa cells among abnormal cells in a sample (either 0 or 1 due to clonality), *ρ* is sample purity (drawn from a uniform distribution), *p* is the proportion of kappa cells in a full sample (i.e. after mixing normal and abnormal cells), *N* is the total number of cells in a sample, and *n*_*κ*_ is the total number of kappa cells in a sample.

*κ*_*n*_, *κ*_*t*_, and *ρ* are unobserved, *p* is a deterministic function of these, and both *N* and *n*_*κ*_ are observed. Cells were defined as kappa or lambda based on whether they have higher expression of *IGKC* or *IGLC2*, respectively. *µ* and σ^2^ are empirically estimated from the NBM samples in our cohort. Since we do not assume prior knowledge about *ρ*, we use an uninformative Beta(1,1) (i.e., uniform) prior. We assume that a patient is equally likely to have a kappa or lambda myeloma (thus the Bernoulli(0.5) distribution for *κ*_*t*_).

We calculate the posterior probability of the sample purity, *ρ*, given the number of kappa cells and the total number of cells in the sample:

2 $${{{=P}}}\left({{{{\rho }}}} | {{{{n}}}}_{{{\kappa }}},{{{N}}}\right)\propto {{{P}}}\left({{{{\rho }}}} | {{{N}}}\right){{{P}}}\left({{{{n}}}}_{{{\kappa }}} | {{{{\rho }}}},{{{N}}}\right)$$

3 $$=P(\rho | N)\mathop{\sum }\limits_{{\kappa }_{t}\in 0,1}{\int }_{0}^{1}P({n}_{\kappa },{\kappa }_{n},{\kappa }_{t} | \rho,N)d{\kappa }_{n}$$

4 $$={{{P}}}({{{{\rho }}}} | {{{N}}})\mathop{\sum }_{{{{\kappa }}}_{{{{t}}}}\in 0,1}{\int }_{0}^{1}{{{P}}}({{{{n}}}}_{{{\kappa }}}{ | {{\kappa }}}_{{{{n}}}},{{{\kappa }}}_{{{{t}}}},{{{{\rho }}}},{{{N}}}){{{P}}}({{{\kappa }}}_{{{{n}}}},{{{\kappa }}}_{{{{t}}}} | {{{{\rho }}}},{{{N}}}){{{{{{\rm{d}}}}}}{{\kappa }}}_{{{{{{\rm{n}}}}}}}$$

5 $$=P(\rho )\mathop{\sum}\limits_{{\kappa }_{t}\in 0,1}{\int }_{0}^{1}P({n}_{\kappa } | {\kappa }_{n},{\kappa }_{t},\rho,N)P({\kappa }_{n})P({\kappa }_{t})d{\kappa }_{n}$$

In the above set of equations, (2) is by Bayes’ rule, (3) is due to the marginalization over κ_*n*_ and κ_*t*_, (4) is simply the factorization of a joint probability, and (5) uses the assumption that *ρ, κ*_*n*_ and *κ*_*t*_ are independent of each other and of *N*.

In our implementation, we normalize this function numerically by calculating the probability of 100 equally spaced values for *ρ* in [0,1]. We report the mode of *P*(*ρ*) as the purity estimate, along with 95% confidence intervals, calculated as the values corresponding to 2.5% and 97.5% of the cumulative distribution.

We include a schematic that visualizes the generative model in Supplementary Fig. 5. A Python implementation of this purity model is available on GitHub (see Code availability).

### Sample clustering approach to labeling normal and abnormal CD138+ cells

For each sample, we performed a cluster analysis of only the cells in that sample. More specifically, we calculated variable genes based on log normalized expression using the highly_variable_genes function from Scanpy^58^ (version 1.7.1) with parameter min_disp=0.6, centered and scaled the data, clipping the resulting values to a maximum of 10, and ran PCA and Leiden clustering (determining the number of PCs to input to Leiden clustering based on an elbow plot). We manually inspected the resulting clusters for each sample to determine whether each cluster contained normal or abnormal cells. This determination was based on whether the cluster uniquely expressed the clonal immunoglobulin for that tumor^59^ (since immunoglobulins were removed from the highly variable gene list, they did not influence the clustering results), as well as each cluster’s expression of certain oncogenes, such as *CCND1* for t(11;14) tumors. All cells were labeled in this way, except for 20 cells from sample MGUS-2 that were characterized by low expression of *MALAT1* and were not obviously similar to the normal or abnormal cells from that sample, and thus were not classified and were excluded from downstream analyses. See Supplementary Fig. 3 for an example of this method applied to a sample.

In addition to labeling each individual cell as normal or abnormal, this approach allowed us to determine a tumor purity for each sample, i.e. the fraction of cells labeled abnormal. To calculate confidence intervals on this purity estimate, we assumed that our observed data was generated as *n ~* Binomial(*N,p*), where *N* is the total sequenced cells in a sample, *n* of which we labeled as abnormal, and *p* is the proportion of abnormal cells in the patient sample (not just the ones we sequenced). We further assume a uniform prior on *p* (*p ~* Beta(1,1)), thus the posterior distribution on *p* is *p*|*n,N ~* Beta(*n* + 1*,N* − *n* + 1), by conjugacy of the Beta and Binomial distributions. We derived 95% confidence intervals on each sample’s purity estimate based on the inverse cdf of its Beta-distributed posterior.

### Abnormal vs. normal differential expression testing with limma

DEGs between abnormal and normal cells were derived using limma version 3.42.2 with voom transformation^22,23,60^. Samples were split into their abnormal and normal populations, and we refer to each of these as a “pseudosample.” Counts across cells in a pseudosample were summed and used as input to the limma-voom pipeline. Immunoglobulin genes, genes with counts per million (CPM) <5 in all samples, and genes expressed in <5% of both abnormal and normal cells, respectively, were removed prior to analysis, resulting in normalization and DE testing of 6,521 genes. Pseudosamples were normalized using the trimmed mean of M values (TMM) method^61^ and fold changes were calculated as implemented in limma. We controlled for age, sex, sample preparation batch, and whether the sample was fresh or frozen. Age information was missing for one NBM sample, and we filled it using mean imputation based on the ages of the other NBM samples. DEGs were those with a Benjamini-Hochberg FDR<0.1 and |log fold change| > log(1.5).

### Within-patient differential expression testing

For each sample with both abnormal and normal cells detected, we calculated DEGs between their abnormal and normal cell populations using a Wilcoxon rank sum test, correcting for multiple hypothesis testing across genes tested for each patient. We calculated fold changes as implemented in Scanpy^58^ (version 1.7.1), but replacing their offset term of 1 × 10^−9^ with half of the minimum (non-zero) log-normalized expression value in our data (0.126), to avoid inflating fold changes. Specifically, fold change was calculated as the ratio of

6 $${{\exp }}\left[\frac{1}{N}{\sum }\left(\mbox{log-normalized expression}\right)-1\right]+{{{{{\rm{offset}}}}}}$$

in each group, where *N* denotes the number of cells in the group. Differentially expressed genes were those with a Benjamini-Hochberg FDR<0.1 and |log fold change| > log(1.5).

For visualizing DEGs uniquely found using this within-patient DE approach (Fig. 2g), we first limited DEGs to those not found using limma. Then, for each gene, we calculated a maximum log_2_(q-value) as the maximum (BH-corrected) q-value reported across patients, multiplied by the number of patients with DEGs (10, in our data) to further correct for multiple hypothesis testing across patients. This value was calculated separately for upregulated and downregulated instances of DEGs, where applicable.

### Automatic relevance determination nonnegative matrix factorization (ARD-NMF) gene expression signatures

We defined gene expression signatures using our SignatureAnalyzer-GPU tool^40^ (see Code availability), which implements a previously described ARD-NMF algorithm^18^. This method approximates the gene expression profile of each cell (represented as a column in the genes-by-cells input matrix, **V**) as an additive combination of latent gene expression signatures (each column in the genes-by-signatures **W**-matrix), each with an associated weight or ‘activity’ in each cell given by the signatures-by-cells **H**-matrix:

7 $${{{{{{\bf{V}}}}}}}\approx \hat{{{{{{{\bf{V}}}}}}}}\equiv {{{{{{\bf{WH}}}}}}}$$

This Bayesian variant of NMF encourages sparse interpretable solutions by imposing either exponential or half normal priors on the weights of the **W**- and **H**-matrices and allows automatic discovery of the number of signatures (*K*) required to explain the data. It solves for the **W** and **H** matrices using maximum a posteriori (MAP) estimation over ${{{P}}}\left({{{{{{\bf{W}}}}}}},{{{{{{\bf{H}}}}}}},{{{{{\boldsymbol{\lambda }}}}}},|,{{{{{{\bf{V}}}}}}}\right)$, where ${{{{{\boldsymbol{\lambda }}}}}}$ is a vector of signature relevance weights. Using a Poisson noise model for our data, an exponential prior on **W**, and a half-norm prior on **H**, the objective function for ARD-NMF described in equation 19 of the original paper^18^ is given by:

8 $$\log {{{P}}}({{{{{{\bf{W}}}}}}},{{{{{{\bf{H}}}}}}},{{{{{\boldsymbol{\lambda }}}}}} | {{{{{{\bf{V}}}}}}})=	{{{{{{\rm{D}}}}}}}_{{{{{{\rm{KL}}}}}}}({{{{{{\bf{V}}}}}}} | {{{{{{\bf{W}}}}}}},{{{{{{\bf{H}}}}}}})\\ 	+\mathop{\sum }\limits_{{{{k}}}=1}^{{{{K'}}} }\frac{1}{{\lambda }_{{{{k}}}}}\left(\mathop{\sum }\limits_{{{{g}}}=1}^{{{{G}}}}{{{{w}}}}_{{{{g}}},{{{k}}}}+\mathop{\sum }\limits_{{{{c}}}=1}^{{{{C}}}}\frac{{{{{h}}}}_{{{{k}}},{{{c}}}}^{2}}{2}+{{{b}}}\right)\\ 	+\left({{{G}}}+\frac{{{{C}}}}{2}+{{{a}}}+1\right)\log {\lambda }_{{{{k}}}}+{{{{{\rm{cst}}}}}}({{{a}}},{{{b}}})$$

where D_KL_ is the Kullback–Leibler divergence, *g* represents a given gene out of *G* total genes, *c* represents a given cell out of *C* total cells, *a* and *b* are hyperparameters (how we chose *a* and *b* is described below), *λ*_*k*_ is a learned relevance weight for signature *k*, *w* and *h* represent elements from the **W** and **H** matrices respectively, and cst(*a*,*b*) is a constant that depends only on *a* and *b*.

After signature discovery, the columns of **W** were normalized to a sum of 1 and all the weight was shifted into the **H**-matrix:

9 $${{{{w}}}}_{{{{g}}},{{{k}}}}\leftarrow \frac{{{{{w}}}}_{{{{g}}},{{{k}}}}}{{\sum }_{{{{{g'}}}} = 1}^{{{{G}}}}{{{{w}}}}_{{{{{g'}}}} ,{{{k}}}}}$$

10 $${{{{h}}}}_{{{{k}}},{{{c}}}}\leftarrow \left(\mathop{\sum }_{{{{g}}} = 1}^{{{{G}}}}{{{{w}}}}_{{{{g}}},{{{k}}}}\right){{{{h}}}}_{{{{k}}},{{{c}}}}$$

for gene *g* (out of *G* total genes), signature *k* and cell *c*.

Our input data was UMI counts for 3883 highly variable genes (dimensions of **V** = 3883 × 29,387), which were determined using the highly_variable_genes function from Scanpy^58^ (version 1.7.1) with min_disp=0.2, which we set to be lower than the default value in order to include genes which may have relevance to plasma and myeloma cell biology despite a modest dispersion value. In addition to other default settings for the SignatureAnalyzer-GPU tool, we used a Poisson objective with an L1 prior on **W** and an L2 prior on **H**, set the initial *K* to 50, the maximum number of iterations to 7000, and the tolerance to 1 × 10^−5^. Following the guidelines in the original ARD-NMF paper^18,40^, we set hyperparameter *a* = 10 (the default in SignatureAnalyzer-GPU) and then calculated *b* as a function of *a*, as implemented by SignatureAnalyzer-GPU. We held out 20% of cells as a validation set. Since the ARD-NMF algorithm finds a local minimum each time it is run, we ran the algorithm 100 times on our data in order to choose an optimal solution. Over 100 runs, the algorithm returned solutions with *K* between 24 and 30 with a mode of 28, and we chose the set of signatures with the lowest beta divergence over the validation set from among the solutions with *K* = 28 (dimensions of **W** = 3883 × 28; **H** = 28 × 29,387). Before analyzing the signature results, we normalize each column in **H** by that cell’s total counts.

A signature was classified as “patient-specific” if its mean activity across cells in any one patient was >4 standard deviations higher than in all other patients. Otherwise, a signature was classified as “single-gene” if the weight of its most highly weighted gene based on the **W** matrix was ≥0.5 more than the weight of its next highest weighted gene. If a signature doesn’t meet either of these criteria, we describe it according to its top genes, where signature genes are ranked by their weight in **W** multiplied by their specificity to that signature, with specificity *s* defined as:

11 $${{{{s}}}}_{{{{g}}},{{{k}}}}=\frac{{\left[{{{{{{\bf{H}}}}}}}\cdot {{{\bf{1}}}}\right]}_{{{{k}}}}{{{{w}}}}_{{{{g}}},{{{k}}}}}{{\sum }_{{{{{k'}}} }=1}^{{{{{k'}}}}}\left({\left[{{{{{{\bf{H}}}}}}}\cdot {{{\bf{1}}}}\right]}_{{{{{k'}}}}}{{{{w}}}}_{{{{g}}},{{{{k'}}}}}\right)}$$

Signatures significantly altered between disease states were identified by calculating the mean signature activity for the abnormal and normal cell populations in each sample, respectively, and performing a Kruskal–Wallis one-way analysis of variance and Dunn’s multiple comparison test with Bonferroni correction to detect differences in mean activities between the following groups: NBM, normal MGUS, normal SMM, normal MM, abnormal MGUS, abnormal SMM, and abnormal MM. Comparisons with family-wise error rate < 0.1 were considered significant.

We additionally ran ARD-NMF on an external single cell dataset^15^ using the same methods as above. We first limited the external data to cell types and disease stages which are present in our data, retaining only bone marrow PCs derived from healthy donors and patients with MGUS, SMM or MM, and then limited the input features to hypervariable genes across these cells (4,669 genes).

### Testing to ensure that signature activity did not correlate with batch variables

For our NMF analysis, we interrogated whether the activity level of any signature is correlated with batch variables (age, sex, sample preparation batch, and fresh/frozen storage). To do this, we limited our data to normal plasma cells, for which we would not expect to find significant differences in signature activity between samples, and then tested for differences in distributions of signature activities between samples from the different batch groups (testing each batch variable separately; using a rank sum test for sex and fresh/frozen, a Kruskall Wallis rank test for batch, and Pearson and Spearman correlations for age). None of the batch variables were significantly correlated with signature activity, using *p* < 0.05 as the significance threshold. Given that we did not observe batch-related differences in signature activity in our normal plasma cells, we conclude that the differences that we observed between abnormal samples at different disease stages are indeed driven by the disease.

### Estimating normal plasma cell signature activity in the MMRF dataset

To estimate the activity of a gene expression signature for each sample in the publicly available MMRF bulk RNA-sequencing dataset (https://research.themmrf.org), we: calculated log-normalized transcripts per million (tpm) on the MMRF counts data using the DESeq2 method for size factors, where samples are normalized using the median, across genes, of the ratios of gene counts to each gene’s geometric mean across samples^62^. Then, for top signature genes (for our normal plasma cell signature, these included *CD27*, *CD79A*, *RNU12*, *JSRP1*, *SAT1*, *CTSH*, and *HCST*), we z-scored the log-tpm expression of each gene across samples, and calculated the signature activity as the mean of z-scored gene expression values.

### Pseudobulking procedure

To pseudobulk samples, we summed the gene counts across cells, calculated the total gene counts in the sample (ignoring genes that accounted for >5% of counts), divided the summed count vector by the total gene counts, and multiplied by one million.

### Single sample GSEA (ssGSEA)

Samples were split into their abnormal and normal populations, and we refer to each of these as a “pseudosample.” We calculated the pseudobulk expression for each pseudosample and input this to the ssGSEA module available on the GenePattern platform^63,64^ to calculate enrichment scores for the hallmark gene sets provided by the Molecular Signature Database (MSigDB)^46^. We removed pseudosamples comprised of <20 cells from downstream analysis of ssGSEA results, due to the high variance inherent in their gene expression. Differential pathway activity between two groups of pseudosamples was calculated using a t-test, and pathways with BH FDR < 0.1 were reported.

### Assessing intratumor heterogeneity for NMF signatures

For each sample, we limited our analysis to abnormal cells and used Scanpy’s^58^ (version 1.7.1) built-in functions to compute highly variable genes (min_mean = 0.0125, max_mean = 3, min_disp=0.6; genes located on immunoglobulin loci were removed), scale the data (max_value=10), compute the 10 first principal components, compute a neighborhood graph (n_neighbors=15) and run Leiden clustering (resolution = 0.6). Parameters that differed from the Scanpy defaults are shown in parentheses. This defined clusters for each sample. We determined that a sample contained a heterogeneous population of cells vis-a-vis a given signature if the mean activity of that signature had a coefficient of variation > 1 across clusters. Specifically, for a given sample, we calculated the mean activity of a given signature in each cluster, producing a vector of means μ with length equal to the number of clusters. We then considered a sample-signature pair to exhibit intratumor heterogeneity if $\frac{{{\mbox{std}}}\left({{{{{\rm{\mu }}}}}}\right)}{{{\mbox{mean}}}\left({{{{{\rm{\mu }}}}}}\right)} > 1$. Signature activities across sample clusters are shown in Supplementary Fig. 11 for all signature-sample pairs which passed this threshold. Our implementation of this analysis is available on GitHub (see Code availability).

### Statistical analysis

Kruskal–Wallis one-way analysis of variance and Dunn’s multiple comparison test with Bonferroni corrections were used when three or more independent groups were compared. When comparing two independent groups, all parametric tests were two-tailed, and the Benjamini-Hochberg (BH) method was used to correct for multiple hypothesis testing where appropriate. *P* < 0.05 or *q* < 0.1 (in cases of multiple hypothesis correction) were considered statistically significant. Error bars plotted on visualizations of mean signature activity or gene expression in a sample represent the standard error of the mean and were calculated as the standard deviation of the means of 10,000 bootstrapped versions of that sample.

### Reporting summary

Further information on research design is available in the Nature Portfolio Reporting Summary linked to this article.

## Supplementary information

Supplementary Information

Reporting Summary

Description of Additional Supplementary Files

Supplementary Data 1

Supplementary Data 2

Supplementary Data 3

Supplementary Data 4

Supplementary Data 5

Supplementary Data 6

Supplementary Data 7

Supplementary Data 8

## Data Availability

The scRNA-seq data generated in this study have been deposited in the NCBI Gene Expression Omnibus (GEO) database under accession number GSE193531. To protect patient privacy, raw data have been deposited in dbGaP under accession number phs001323.v3.p1. Data access can be requested through the dbGaP portal in accordance with their standard guidelines [https://dbgap.ncbi.nlm.nih.gov/aa/wga.cgi?page=login]. The publicly available MSigDB gene sets used in this study are available from the Molecular Signatures Database v7.5.1 [http://www.gsea-msigdb.org/gsea/msigdb]. MMRF CoMMpass data used in this study can be obtained from the MMRF Research Gateway [https://research.themmrf.org]. Source data are provided with this paper.

## Source data

Source Data

## Code availability

The single-cell RNA data was processed using cellranger version 2.0.1 [https://www.10xgenomics.com/] and analyzed with the python package Scanpy version 1.7.1 [https://scanpy-tutorials.readthedocs.io]. Gene expression signatures were extracted using our SignatureAnalyzer algorithm available on GitHub [https://github.com/broadinstitute/SignatureAnalyzer-GPU, commit 4c54ff6690378a0e87bdd8da7041e50d5381443f]. Differential expression testing between normal and neoplastic pseudobulk samples was performed in R version 4.1.0 using the limma software version 3.42.2 [https://bioconductor.org/packages/release/bioc/html/limma.html]. Other analysis code is available at https://github.com/getzlab/Boiarsky-etal-2022.
