# Supplementary material for: Single cell characterization of myeloma and its precursor conditions reveals transcriptional signatures of early tumorigenesis
Source: Nat Commun. 2022 Nov 17;13:7040. doi: 10.1038/s41467-022-33944-z (PMC9672303; doi:10.1038/s41467-022-33944-z)
Supplement: Supplementary file 1 — Supplementary Information [file 41467_2022_33944_MOESM1_ESM.pdf]

Supplementary Information

Supplementary Note 1

While the cells in cluster 17 passed our QC pipeline and were therefore included in the analysis, cells in this cluster are characterized by extremely low expression of *MALAT1*, as well as a low number of genes detected overall (median number of genes detected is lowest in cluster 17 compared to all other clusters), likely signifying a cell quality issue. Clusters 11 and 19 each grouped cells with no specific translocation and a relatively low number of abnormal cells sequenced.

Supplementary Note 2

Two gene signatures discovered in the CD138+ cells involved interferon-inducible genes: W24 (the plasma cell "IFN inducible" signature), whose activity is shown in the topmost heatmap in Fig. 4a, and W7, a patient-specific signature which involves many varied genes highly expressed in sample MM-4, including *IFI27* and *IFI6*. Because W7 also involves non-interferon related genes, we do not show it in the main figure, but MM-4's high expression of interferon-inducible genes is captured by that signature (mean activity of W7 in MM-4 = 276) instead of W24.

Supplementary Data

Supplementary Data 1. A mapping between the sample IDs for the CD138+ cells analyzed in this study and the corresponding sample IDs for the CD138- cells analyzed in Zavidij et al.[1].

Supplementary Data 2. Patient data and clinical information. List of clinical measurements for samples used for single-cell RNA sequencing, including age, disease_stage, sex, race, Type (type of the immunoglobulin involved in myeloma), 1st FISH results (cytogenetic results from iFISH at timepoint 1), 2nd FISH results (cytogenetic results from iFISH at timepoint 2), time from

dx to 1st FISH results (time of 1st iFISH assay, in days from diagnosis), time from dx to 2nd FISH results (time of 2nd iFISH assay, in days from diagnosis), M Protein when sample was taken (g/dL), BMPC % (% plasma cells in bone marrow biopsy), serum free light chain ratio (involved/uninvolved), 20/2/20 risk for SMM patients[2], progression_to_mm (1=Has progressed to MM, 2=Has not progressed to MM, 3=MM was the original diagnosis), days till mm diagnosis (from the time of initial diagnosis), treated during MGUS/SMM (0=no, 1=yes), and follow up time per patient (days). **Supplementary Data 3. Sample information.** Quality metrics for scRNAseq samples, including whether the sample was fresh or frozen, batch ID, n cells retained after removing low quality cells and non-CD138+ cells (QC), and median UMI post-QC. **Supplementary Data 4. Cell level information.** Sample of origin, Leiden clustering assignment, normal/abnormal label, n genes detected, fraction mitochondrial reads, and n UMI detected per cell, for cells retained post-QC. **Supplementary Data 5. DEGs between CD20+ and CD20- subclones in SMM-12.** List of differential expression testing results for cells in SMM-12’s CD20+ vs. CD20- subclones. A two-sided Wilcoxon rank sum test with Benjamini-Hochberg correction was used, and fold changes were calculated as described in Methods (“Within-patient differential expression testing”). **Supplementary Data 6.** List of 764 differentially expressed genes (|log(fold change)| > log(1.5); q<0.1) discovered for abnormal vs. NBM samples using limma-voom[3,4]. For each gene, we report the logFC ($\log_2$ fold change of abnormal/NBM), AveExpr (average expression across all samples, in $\log_2$ CPM), t (logFC divided by its standard error), P.Value (Raw p-value (based on t) that logFC differs from 0), and adj.P.Val (Benjamini-Hochberg false discovery rate adjusted p-value). **Supplementary Data 7.** List of differentially expressed genes (two-sided Wilcoxon rank sum test with Bejamini-Hochberg correction, |log(fold change)| > log(1.5); q<0.1) discovered per-sample using our within-patient differential expression analysis, along with their fold changes and significance levels. Genes will appear more than once if they were significant in multiple samples. **Supplementary Data 8.**

Full list of NMF signatures. List of top genes and descriptions for all 28 signatures discovered using Bayesian NMF.

Supplementary references

1. Zavidij, O. *et al.* Single-cell RNA sequencing reveals compromised immune microenvironment in precursor stages of multiple myeloma. *Nature Cancer* **1**, 493–506 (2020).
2. Lakshman, A. *et al.* Risk stratification of smoldering multiple myeloma incorporating revised IMWG diagnostic criteria. *Blood Cancer J.* **8**, 1–10 (2018).
3. Ritchie, M. E. *et al.* limma powers differential expression analyses for RNA-sequencing and microarray studies. *Nucleic Acids Res.* **43**, e47–e47 (2015).
4. Law, C. W., Chen, Y., Shi, W. & Smyth, G. K. voom: Precision weights unlock linear model analysis tools for RNA-seq read counts. *Genome Biol.* **15**, 1–17 (2014).

Supplementary Figure 1

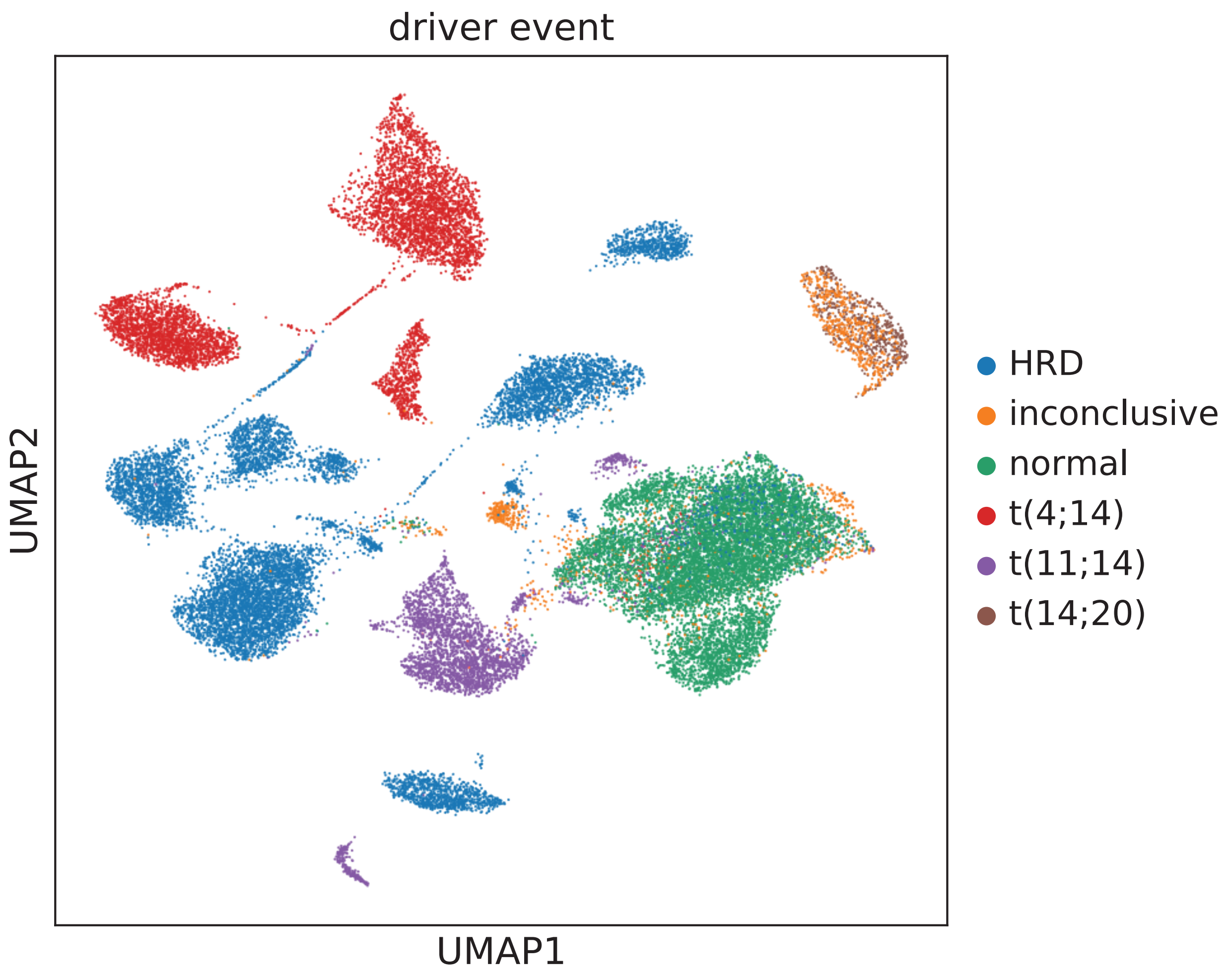

Supplementary Figure 1. Distribution of cancer driver events on UMAP plot

UMAP representation of plasma cells colored by cancer driver event, as determined by iFISH. Driver event is one of: normal, translocation (type specified in legend), hyperdiploid (HRD), or inconclusive test results.

Supplementary Figure 2

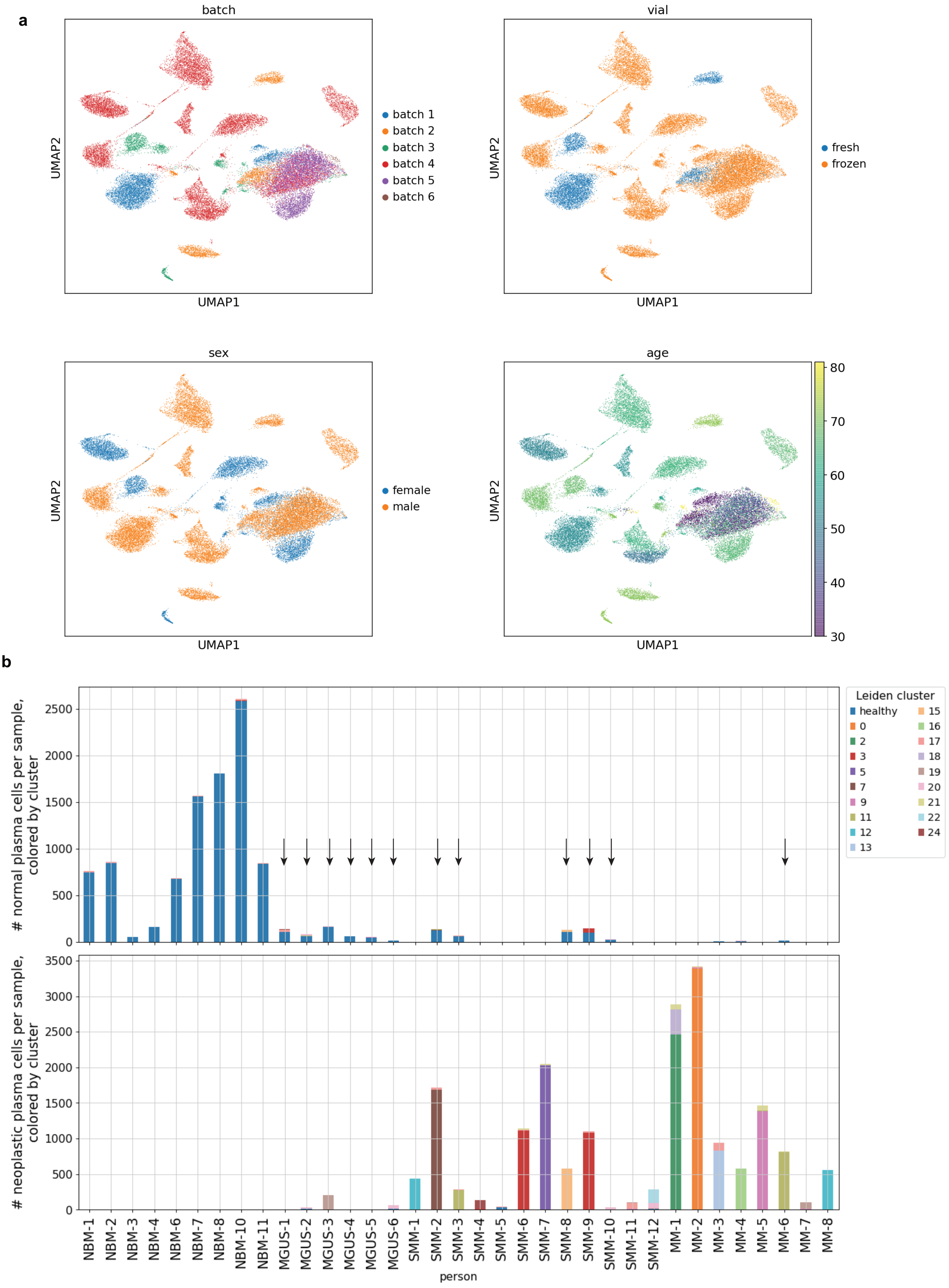

Supplementary Figure 2. Distribution of batch variables across cells and clusters

a, UMAP plot of all CD138+ cells colored by sample preparation batch, vial (whether the cells were fresh or frozen prior to sequencing), sex, and age. **b,** The cluster assignments for cells from each sample, separately for normal cells (top) and abnormal cells (bottom), with normal/abnormal status determined using the per-sample clustering technique described in Methods. Arrows point to normal cells from patients that cluster together with normal cells from NBM, rather than together with the other cells from the same patient donor. This pattern suggests that the disease signal's influence on clustering is stronger than that of a potential batch effect.

Supplementary Figure 3

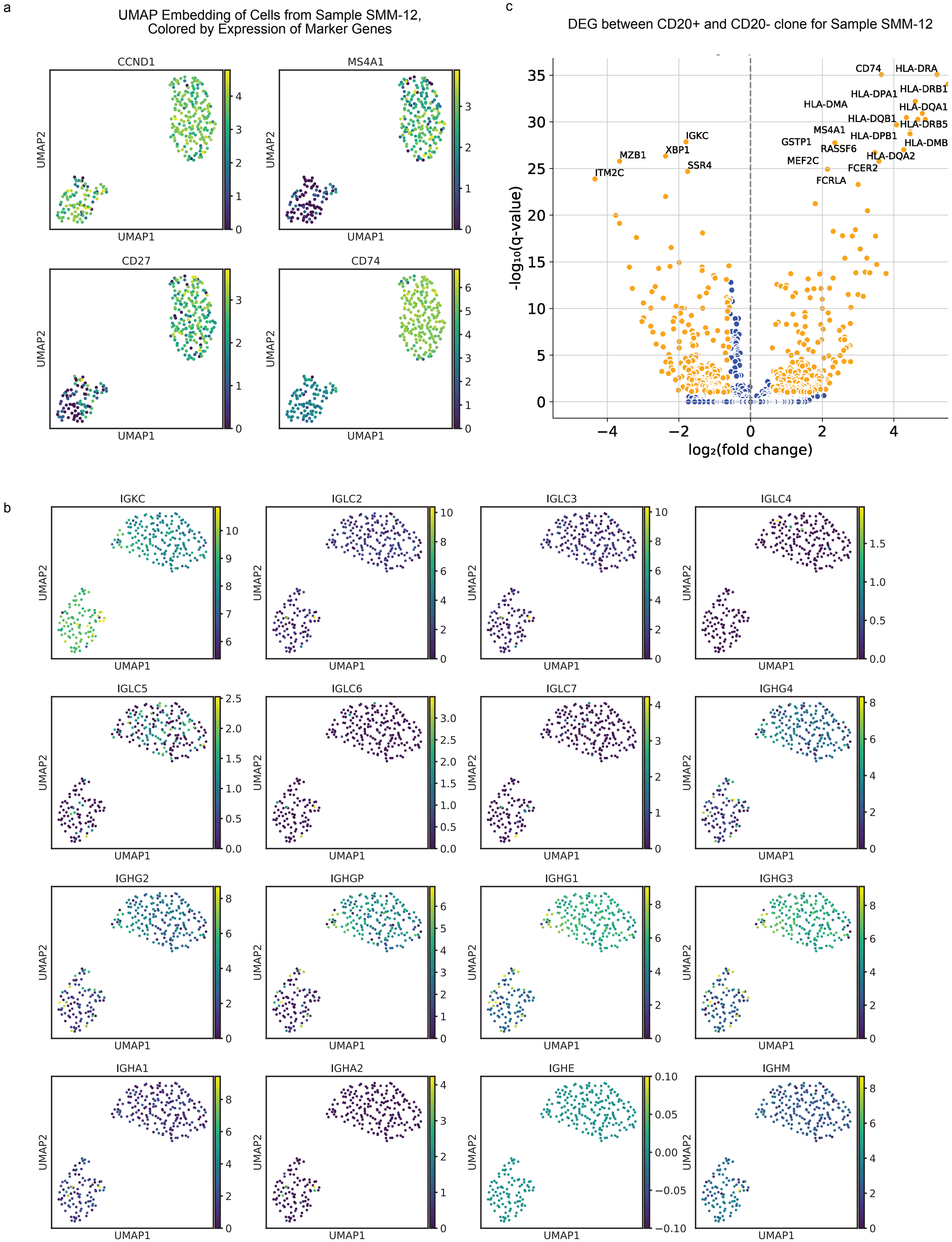

Supplementary Figure 3. Sample SMM-12 has t(11;14) translocation and CD20+ subclone

a, UMAP embedding of cells from sample SMM-12, which has 190 cells belonging to a CD20+ subclone (cluster on right) out of 284 total cells. Both clusters express *CCND1*, signifying that both harbor a t(11;14) translocation, but the cluster on the right expresses higher levels of *CD20 (*also known as *MS4A1), CD27, CD74*. **b,** Volcano plot of DEGs between the CD20+ and CD20- subclones from sample SMM-12. Orange denotes a significant DEG (|log(fold change)| > log(1.5); q<0.1). The top genes, ranked by q-value, are annotated on the plot. **c,** UMAP embedding of cells from sample SMM-12, colored by expression of immunoglobulin constant region genes. Both subpopulations express a kappa light chain (encoded by the gene *IGKC*) and IgG heavy chain (encoded by genes named *IGHG**).

Supplementary Figure 4

a

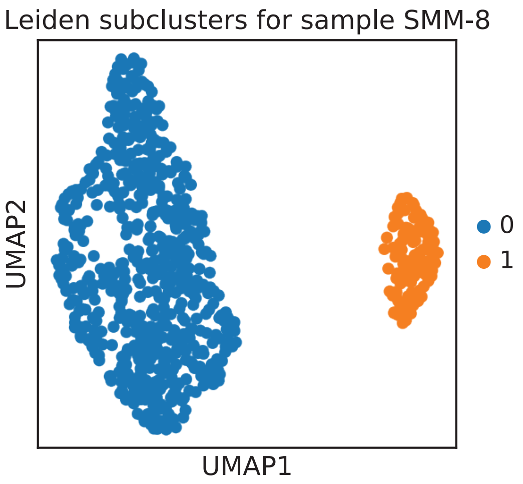

b

Expression of marker genes on cells from sample SMM-8 reveals separation of abnormal and normal plasma cells

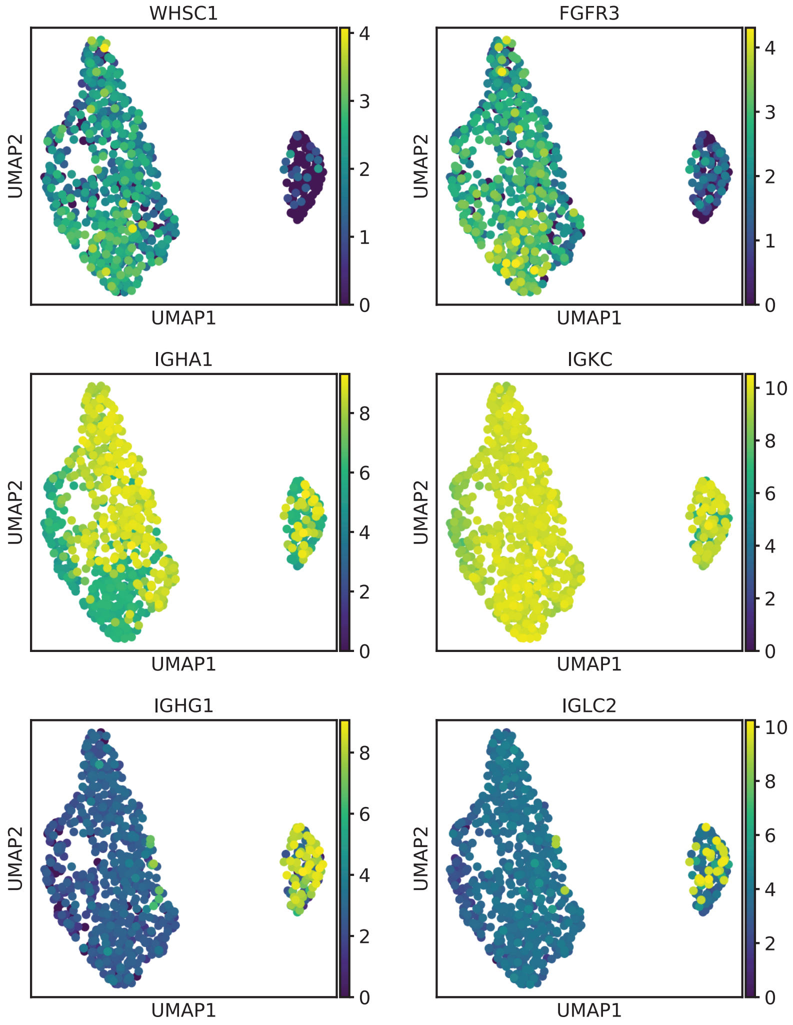

Supplementary Figure 4. Example of clustering approach for labeling normal and abnormal plasma cells within each sample

a, UMAP plot showing clustering for representative sample SMM-8. Genes in immunoglobulin loci were not used in the computation of the UMAP embedding or Leiden clustering. **b,** Expression of genes used to determine that cluster 0 contains abnormal cells and the cluster 1 contains normal cells: t(4;14) translocation associated genes *WHSC1* and *FGFR3* (first row); genes encoding the IgA-kappa immunoglobulin expressed on this patient's monoclonal cells (second row); genes encoding non-clonal immunoglobulin components IgG (*IGHG1*) and the lambda light chain (*IGLC2*) (third row). We observe that cells in cluster 0 clonally express IgA-kappa genes, while cluster 1 contains a mixture of cells expressing IgA and IgG heavy chains and kappa and lambda light chains. We performed a similar analysis for every patient sample in our cohort.

Supplementary Figure 5

Samples are mixtures of an abnormal cell population and a normal cell population.

Each cell in the sample expresses either a kappa (IgK) or lambda immunoglobulin light chain.

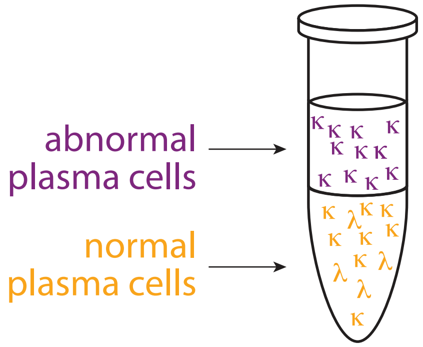

In our Bayesian purity model, a sample is generated based on three prior distributions:

$\rho \sim \mathrm{Beta}(1,1)$

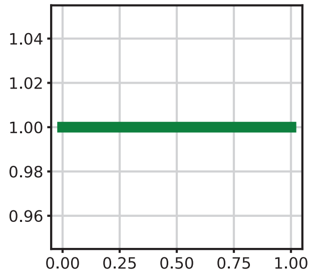

The relative proportion of normal vs. abnormal cells in a sample (i.e. sample purity ρ) is drawn from a uniform prior.

$\kappa_t \sim \mathrm{Bernoulli}(0.5)$

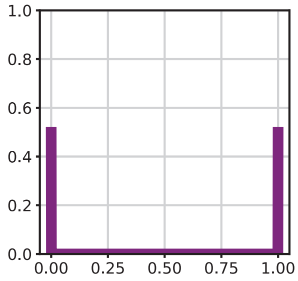

For the abnormal cell population, the proportion of cells expressing IgK is either 0 or 1 (due to clonality), and is drawn is drawn from a Bernoulli distribution with a 50% probability.

$\kappa_n \sim \text{Truncated Normal}(\mu,\sigma^2,0,1)$

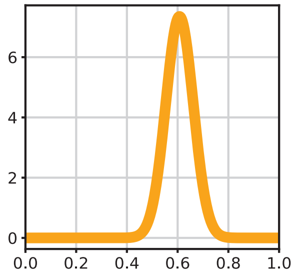

For the normal plasma cell population, the fraction of cells expressing IgK is drawn from a normal distribution truncated between 0 and 1. μ and σ^2 are determined based on the NBM samples in our cohort.

p, the fraction of IgK cells in a sample, is given by:

$$p = \rho(\kappa_t) + (1-\rho)(\kappa_n)$$

...for example, if a sample has purity ρ =1, it will either have 0% or 100% IgK cells, depending on the value of κ_t

Data Likelihood:

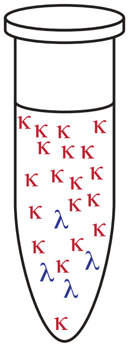

In actuality, we observe a mixture of cells, and we do not know which are normal vs. abnormal. We do observe what immunoglobulin light chain each cell expresses.

The likelihood of observing n_κ kappa-expressing cells in a sample of N cells is modeled as $P(n_\kappa) \sim \mathrm{Binomial}(N, p)$.

Our model uses the likelihood of the observed data and these priors to infer a posterior probability over the purity of each sample.

Supplementary Figure 5. Visual guide to Bayesian purity model

A visual guide to the generative model we assume as part of our Bayesian model for estimating sample purity, to aid in understanding the method.

Supplementary Figure 6

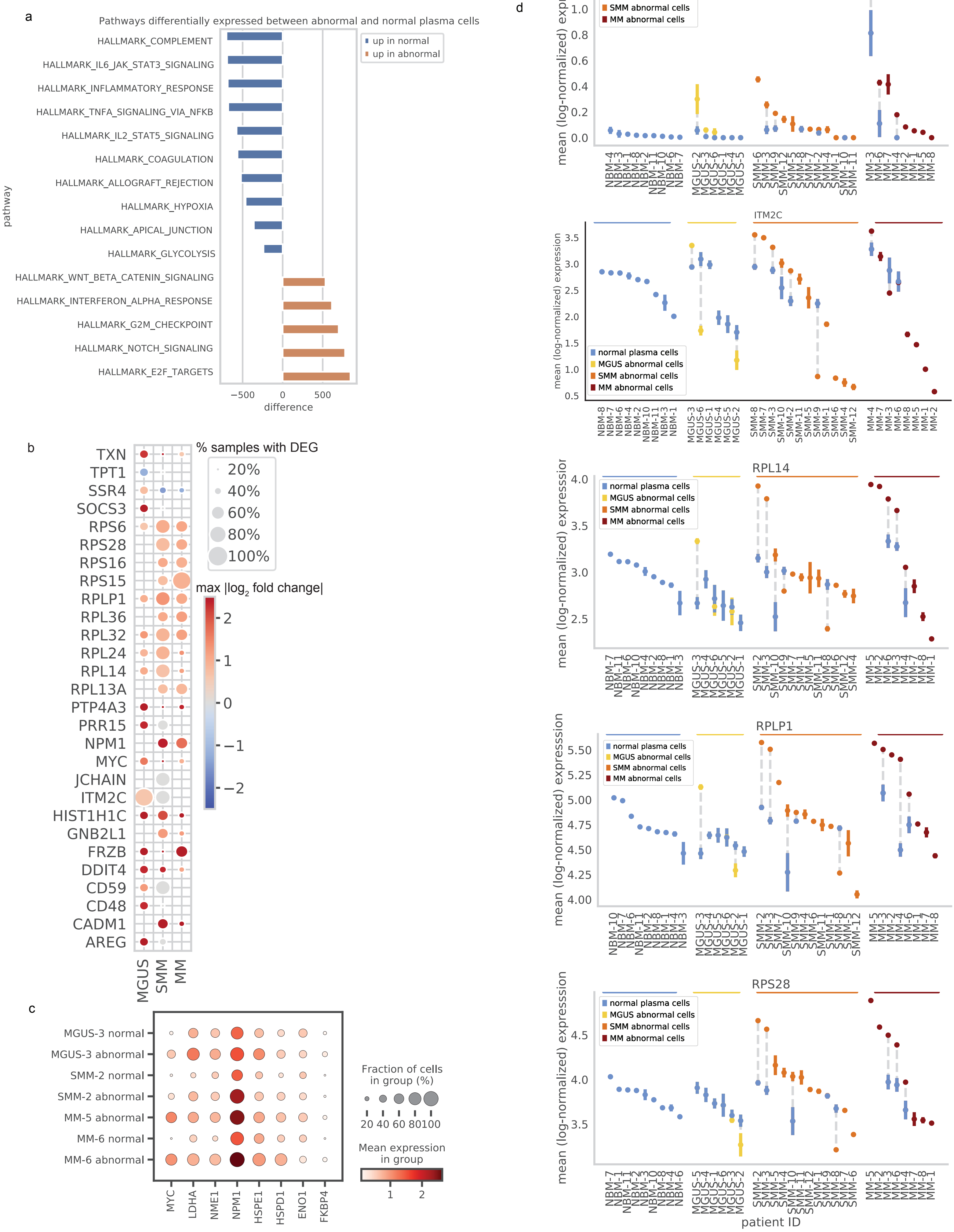

Supplementary Figure 6. Additional visualizations of transcriptional differences detected between normal and abnormal cells

a, MSigDB hallmark genesets differentially enriched in abnormal samples (N=23 independent samples from 22 individuals) compared to normal (N=23 independent samples) (two-sided t-test, q<0.1). The difference between the mean enrichment among abnormal vs. normal samples is plotted on the x-axis. Source data are provided as a Source Data File. **b,** This heatmap portrays how differentially expressed genes (DEGs) were shared or varied across disease stages. The genes shown are the same genes annotated on the volcano plot in Fig. 2g; these genes were uniquely discovered to be differentially expressed using our within-patient DE analysis, and not limma-voom. The size of the circle represents the fraction of samples from each disease stage in which that gene was determined to be differentially expressed (|log(fold change)| > log(1.5); q<0.1), out of a total of two samples that had DEGs from MGUS, five from SMM, and three from MM. The color of the dot represents the direction and magnitude of the greatest significant $|\log_2$ fold change$|$ in each disease stage. For the purposes of the visualization, we cap the color bar at 2.5 and -2.5. **c,** Expression of *MYC* and the MYC activation signature genes reported in Chng et al., 2011 in samples with significant DE of *MYC* by within-patient DE (MGUS-3, SMM-2, MM-6), as well as in MM-5, which was not included in the within-patient DE due to 100% purity, but had high levels of *MYC* comparable to MM-6. Cells are grouped by normal/abnormal labeling as well as sample ID (y-axis). Color intensity corresponds to the mean expression of the gene in each group, and dot size corresponds to the fraction of cells in the group that express the gene. **d,** Mean expression ± s.e.m. of selected DEGs in the normal and abnormal portions of samples at different disease stages.

Supplementary Figure 7

a

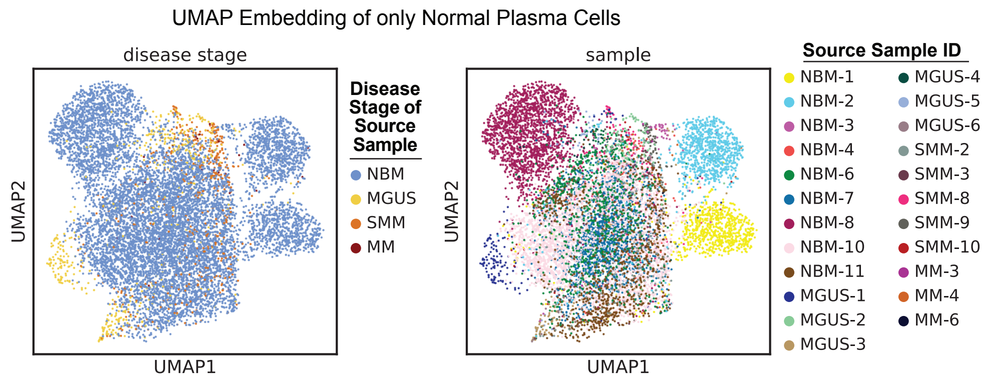

b

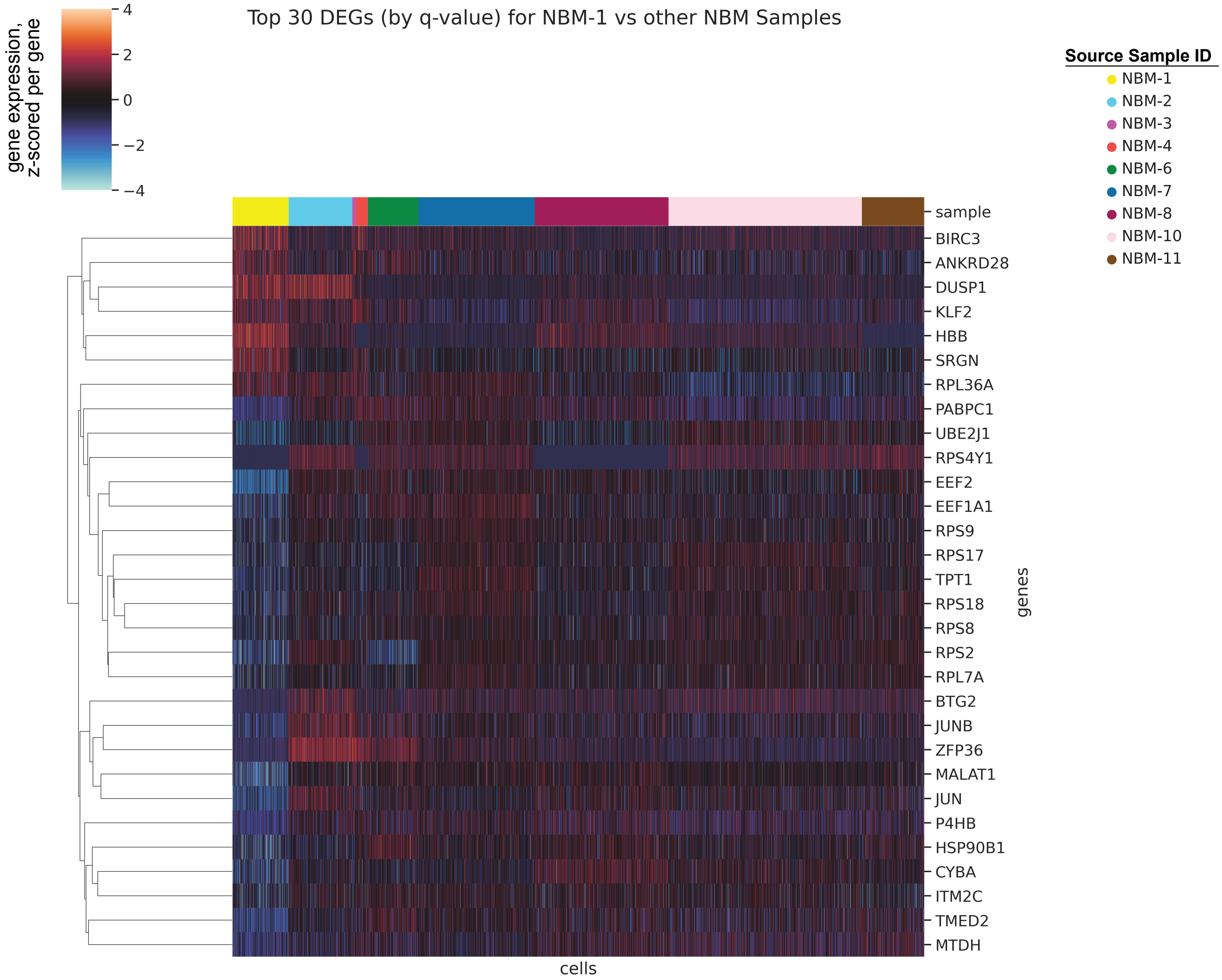

Supplementary Figure 7. Inter-patient variation among healthy cells

a, Inter-patient variation exists even among normal plasma cells (even among those from NBM donors), as observed in these UMAP embeddings containing only cells labeled as normal. The normal plasma cells on these plots are colored by the disease stage (left) and sample ID (right) of the patient from which they were extracted. **b,** As further evidence of inter-patient variation among NBM samples, we found that comparing one NBM sample against the others returns many DEGs. As an example, comparing cells from patient NBM-1 to those from other NBM patients returned 1,061 DEGs (|log(fold change)| > log(1.5); q<0.1). Here, we visualize the top 30 DEGs (by q-value) for NBM-1 vs. other NBMs. Each column represents the gene expression of a single cell, and cells are ordered by the samples from which they originated. Expression is z-scored row-wise.

Supplementary Figure 8

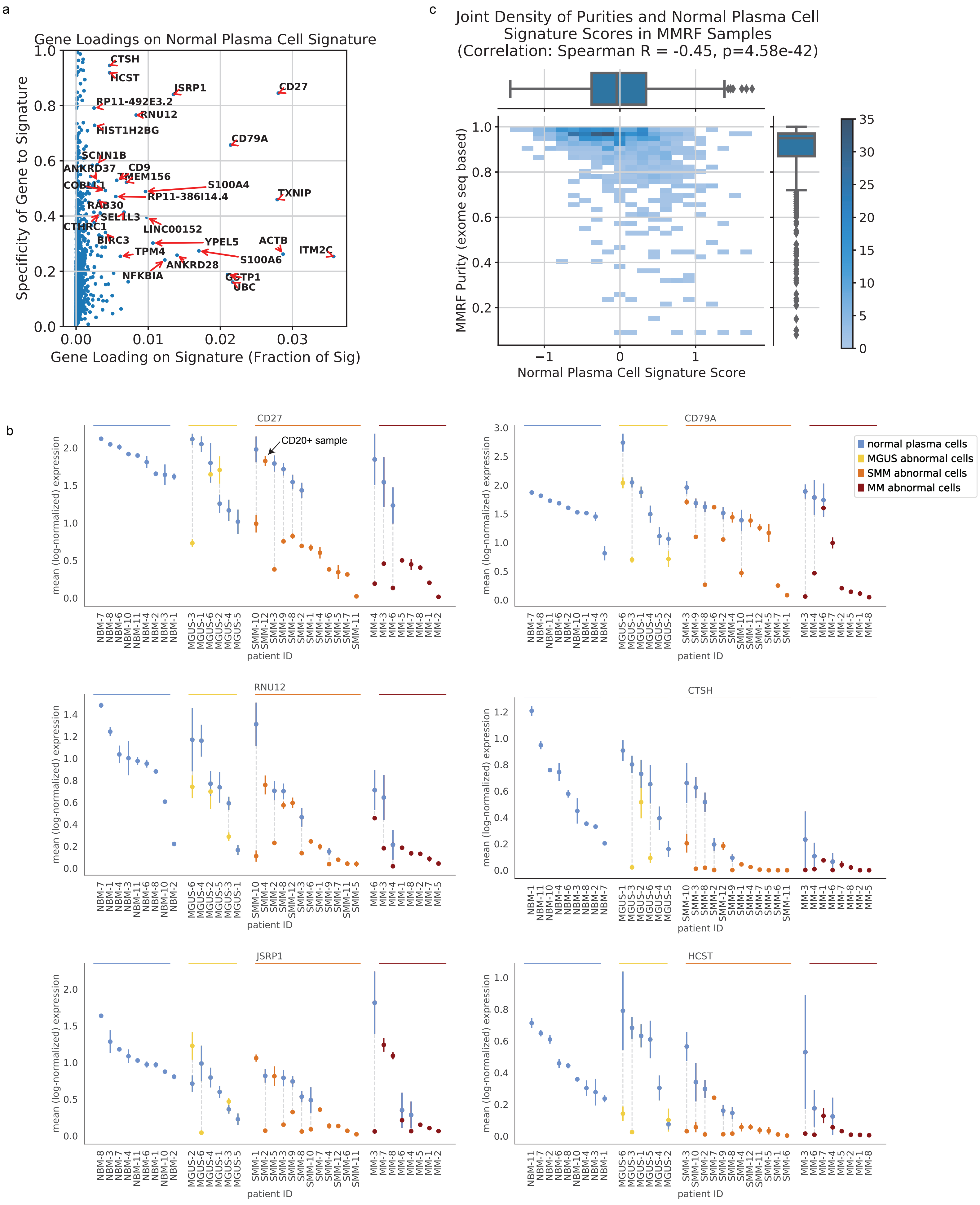

Supplementary Figure 8. Expression of top genes in normal plasma cell signature and validation in bulk MMRF data

a, Contributions of individual genes to the ‘normal plasma cell signature.’ The x-axis represents a gene’s loading on the signature, i.e. its value in the W matrix, and the y-axis represents a gene’s specificity to the signature (see Methods). **b,** Mean expression ± s.e.m. of top genes from the ‘normal plasma cell signature’ in abnormal and normal plasma cell portions of samples. We see that expression of the top genes from the signature are generally also downregulated in abnormal cells as compared to normal plasma cells at all stages of disease. For *CD27*, the one notable exception is sample SMM-12, which has a CD20+ phenotype. **c,** In bulk samples from the MMRF dataset (N=826 independent samples), we observe a significant negative correlation between sample purity and ‘normal plasma cell signature’ score (Spearman R=-0.45, $p=4.58\times10^{-42}$). The joint density of purity and signature score is plotted, with the intensity of color indicating the number of samples at a given part of the distribution (see color bar). Boxplots representing the marginal distributions of signature scores and purities are plotted along the top and right, respectively (center line, median; box limits, upper and lower quartiles; whiskers, 1.5x interquartile range; points, outliers).

Supplementary Figure 9

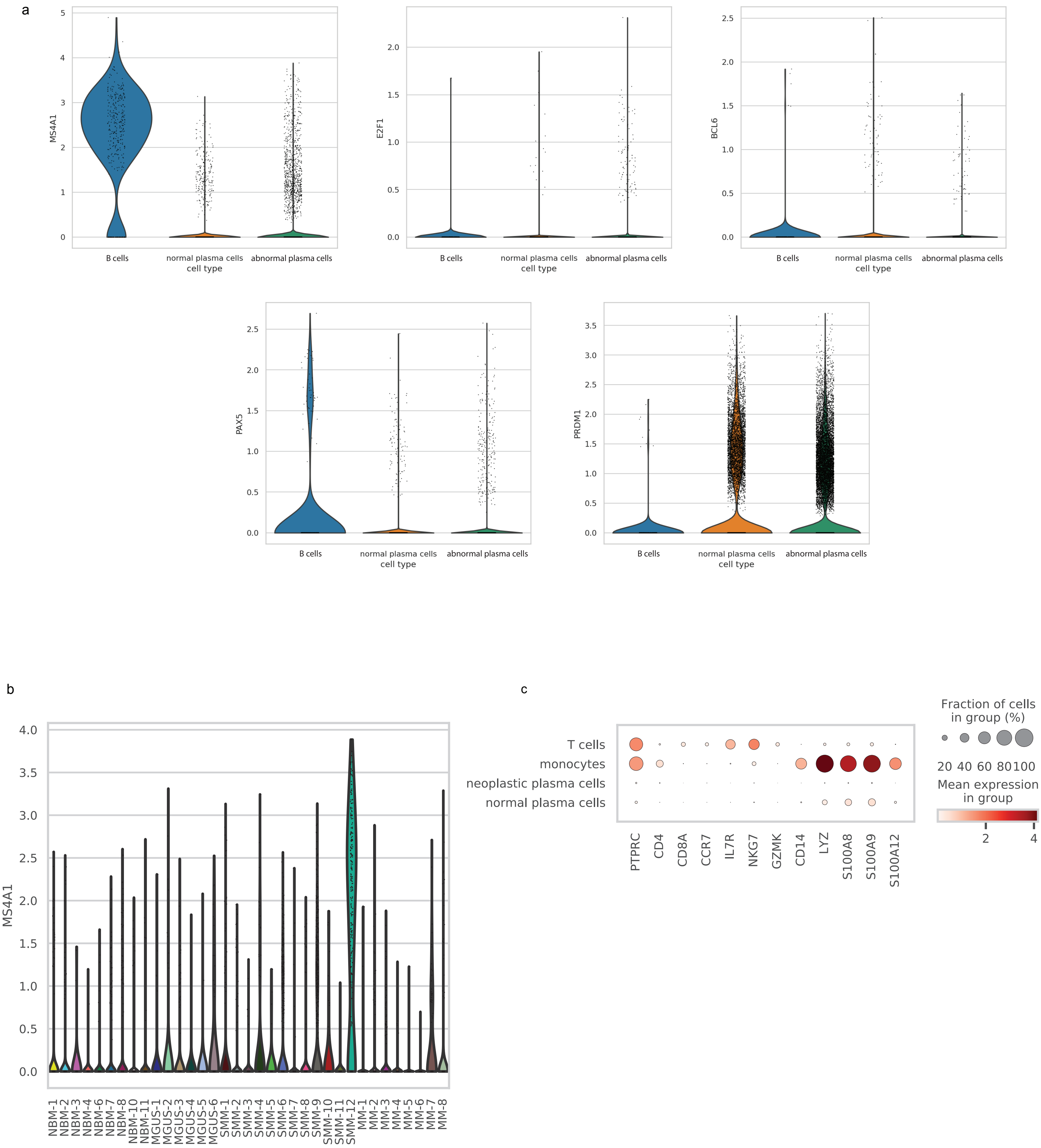

Supplementary Figure 9. No contamination from B cells, T cells, or monocytes in CD138+ cells

a, B cell surface marker *CD20* (also known as *MS4A1)* and B cell transcription factors *E2F1, PAX5,* and *BCL6* are virtually not expressed in normal or abnormal CD138+ cells (with the exception of *CD20* expression in SMM-12; see **(b)**), while they are expressed on the B cells which were removed from our data as part of QC. The plasma cell transcription factor *PRDM1* is expressed on CD138+ cells, but not B cells. Violin plots show distribution of expression over cells in each group. **b,** Expression of *CD20* on CD138+ cells is largely driven by patient SMM-12, who has a CD20+ MM phenotype. **c,** T cell and monocyte marker genes are hardly expressed in our CD138+ normal or abnormal cells, while they are expressed in the T cell and monocyte populations which we removed from our data as part of QC. While there are low levels of monocyte marker genes among normal PCs, indicating possible low levels of ambient contamination in some normal samples, these genes are not expressed in abnormal cells, indicating that abnormal PCs (i.e. the PCs plotted in Figure 4a for comparison with monocytic populations) are uncontaminated. Color intensity corresponds to the mean expression of the gene in each group, and dot size corresponds to the fraction of cells in the group that express the gene.

Supplementary Figure 10

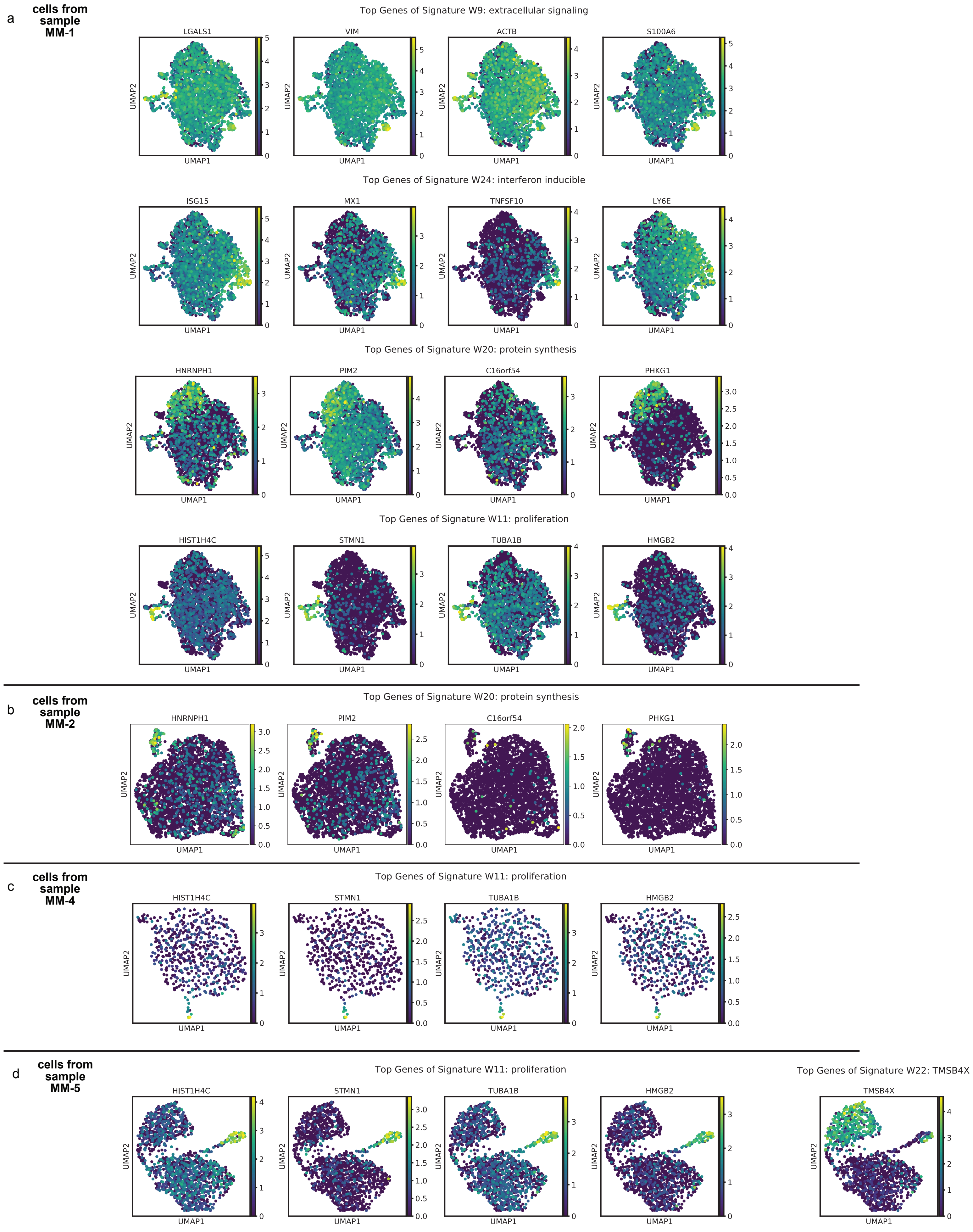

Supplementary Figure 10. Genes are heterogeneously expressed within tumor samples

UMAP embeddings of cells from samples MM-1 **(a)**, MM-2 **(b)**, MM-4 **(c)** and MM-5 **(d)**, colored by expression of the top 4 genes from each signature that was highlighted as heterogeneously active within that patient in Fig. 4c. Since signature 22 was a single-gene signature, we only plotted the expression of the top gene (*TMSB4X)*.

Supplementary Figure 11

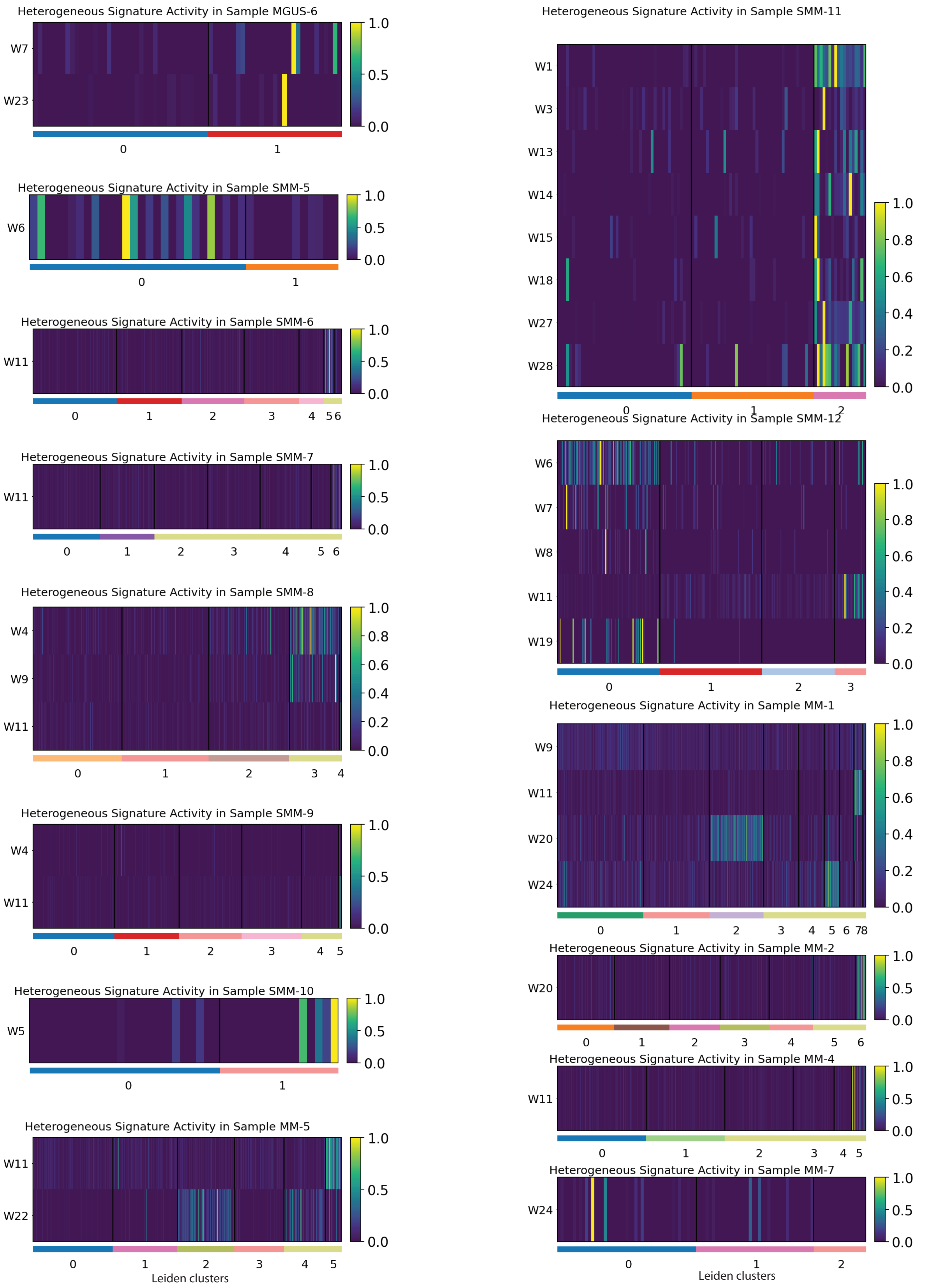

Supplementary Figure 11. NMF signatures exhibit heterogeneous activity across clusters within samples.

Heatmap of NMF signature activity x cells in each sample, for signatures that were found to be heterogeneously active within that sample (see Methods). Rows are standardized (for each row, we subtracted the minimum and divided by its maximum). Cells are ordered according to their cluster assignment (these are the clusters which were used to determine heterogeneous signature activity, as described in Methods; they are distinct from other clusterings mentioned throughout this study).
