# Supplementary material for: Single cell characterization of myeloma and its precursor conditions reveals transcriptional signatures of early tumorigenesis
Source: Nat Commun. 2022 Nov 17;13:7040. doi: 10.1038/s41467-022-33944-z (PMC9672303; doi:10.1038/s41467-022-33944-z)
Supplement: Supplementary file 3 — Description of Additional Supplementary Files [file 41467_2022_33944_MOESM3_ESM.docx]

**Description of Additional Supplementary Files**

File Name: Supplementary Data 1

Description: A mapping between the sample IDs for the CD138+ cells analyzed in this study and the corresponding sample IDs for the CD138- cells analyzed in Zavidij et al.

File Name: Supplementary Data 2

Description: . List of clinical measurements for samples used for single-cell RNA sequencing, including age, disease_stage, sex, race, Type (type of the immunoglobulin involved in myeloma), 1st FISH results (cytogenetic results from iFISH at timepoint 1), 2nd FISH results (cytogenetic results from iFISH at timepoint 2), time from dx to 1st FISH results (time of 1st iFISH assay, in days from diagnosis), time from dx to 2nd FISH results (time of 2nd iFISH assay, in days from diagnosis), M Protein when sample was taken (g/dL), BMPC % (% plasma cells in bone marrow biopsy), serum free light chain ratio (involved/uninvolved), 20/2/20 risk for SMM patients2 , progression_to_mm (1=Has progressed to MM, 2=Has not progressed to MM, 3=MM was the original diagnosis), days till mm diagnosis (from the time of initial diagnosis), treated during MGUS/SMM (0=no, 1=yes), and follow up time per patient (days).

File Name: Supplementary Data 3

Description: Sample information. Quality metrics for scRNAseq samples, including whether the sample was fresh or frozen, batch ID, n cells retained after removing low quality cells and non-CD138+ cells (QC), and median UMI post-QC.

File Name: Supplementary Data 4

Description: Sample of origin, Leiden clustering assignment, normal/abnormal label, n genes detected, fraction mitochondrial reads, and n UMI detected per cell, for cells retained post-QC.

File Name: Supplementary Data 5

Description: DEGs between CD20+ and CD20- subclones in SMM-12. List of differential expression testing results for cells in SMM-12’s CD20+ vs. CD20- subclones. A two-sided Wilcoxon rank sum test with Benjamini-Hochberg correction was used, and fold changes were calculated as described in Methods (“Within-patient differential expression testing”).

File Name: Supplementary Data 6

Description: List of 764 differentially expressed genes (|log(fold change)| *>* log(1*.*5); q<0.1) discovered for abnormal vs. NBM samples using limma-voom[^3,4^](https://paperpile.com/c/TwppJv/Cs9h+sBBW). For each gene, we report the logFC (log2 fold change of abnormal/NBM), AveExpr (average expression across all samples, in log2 CPM), t (logFC divided by its standard error), P.Value (Raw p-value (based on t) that logFC differs from 0), and adj.P.Val (Benjamini-Hochberg false discovery rate adjusted p-value).

File Name: Supplementary Data 7

Description: List of differentially expressed genes (two-sided Wilcoxon rank sum test with Bejamini-Hochberg correction, |log(fold change)| *>* log(1*.*5); q<0.1) discovered per-sample using our within-patient differential expression analysis, along with their fold changes and significance levels. Genes will appear more than once if they were significant in multiple samples.

File Name: Supplementary Data 8

Description:

Full list of NMF signatures. List of top genes and descriptions for all 28 signatures discovered using Bayesian NMF.
